# Supplementary material for: Insights into the Adaptation to High Altitudes from Transcriptome Profiling: A Case Study of an Endangered Species, Kingdonia uniflora
Source: Genes (Basel). 2023 Jun 19;14(6):1291. doi: 10.3390/genes14061291 (PMC10298588; doi:10.3390/genes14061291)
Supplement: Supplementary file 1 [file genes-14-01291-s001.zip › Supplemental Figures.pdf]

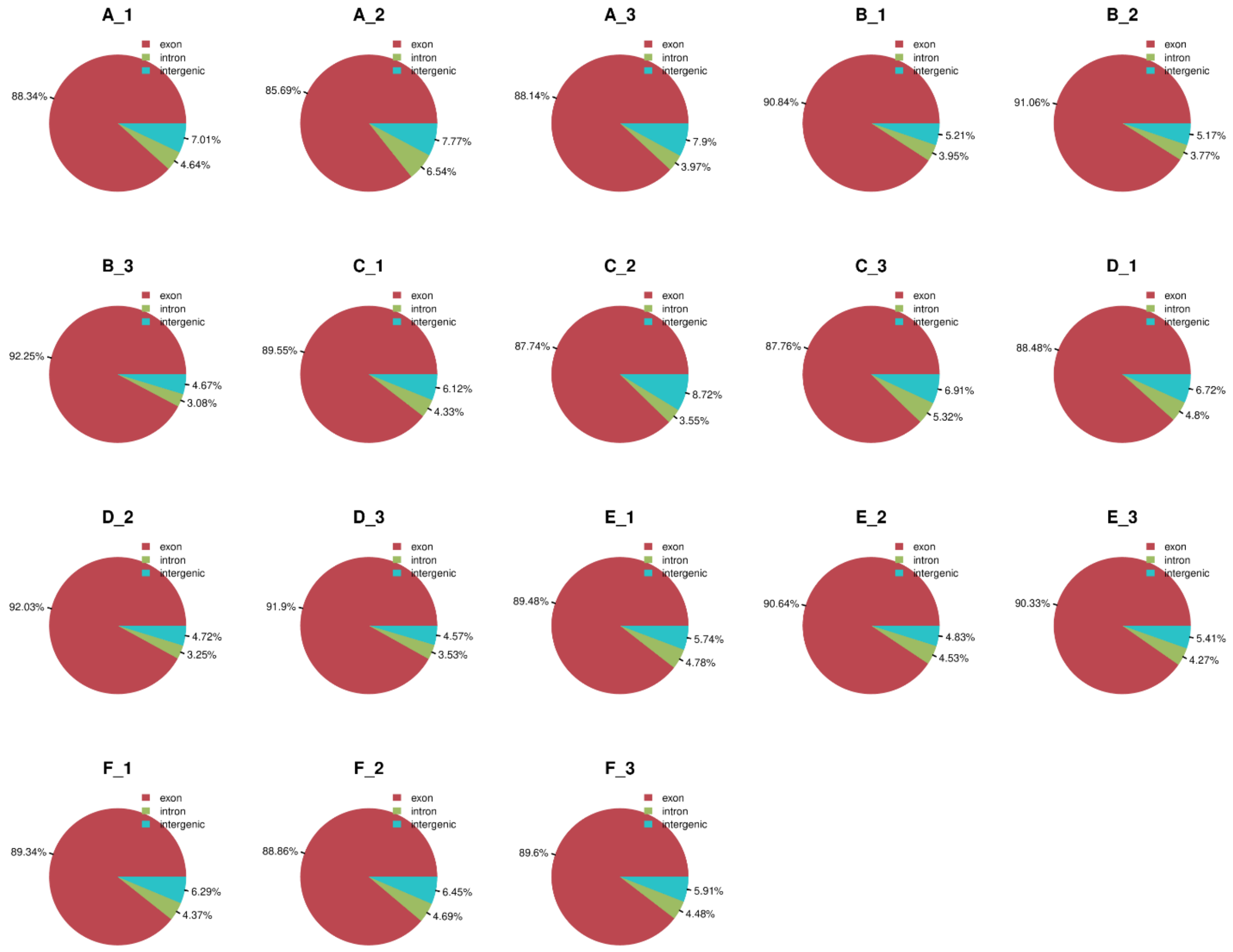

Supplemental Figure S1. The statistics of gene mapped regions in 18 samples.

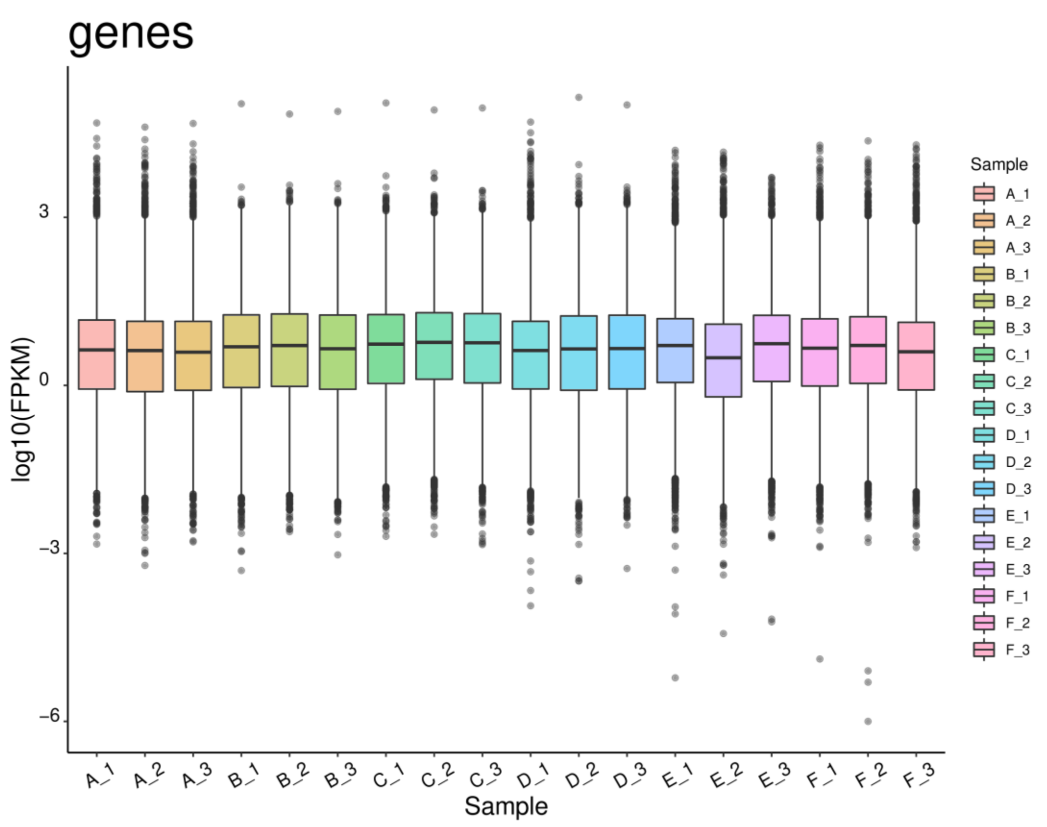
genes
log10(FPKM)
3
0
−3
−6
Sample
A_1
A_2
A_3
B_1
B_2
B_3
C_1
C_2
C_3
D_1
D_2
D_3
E_1
E_2
E_3
F_1
F_2
F_3

a.

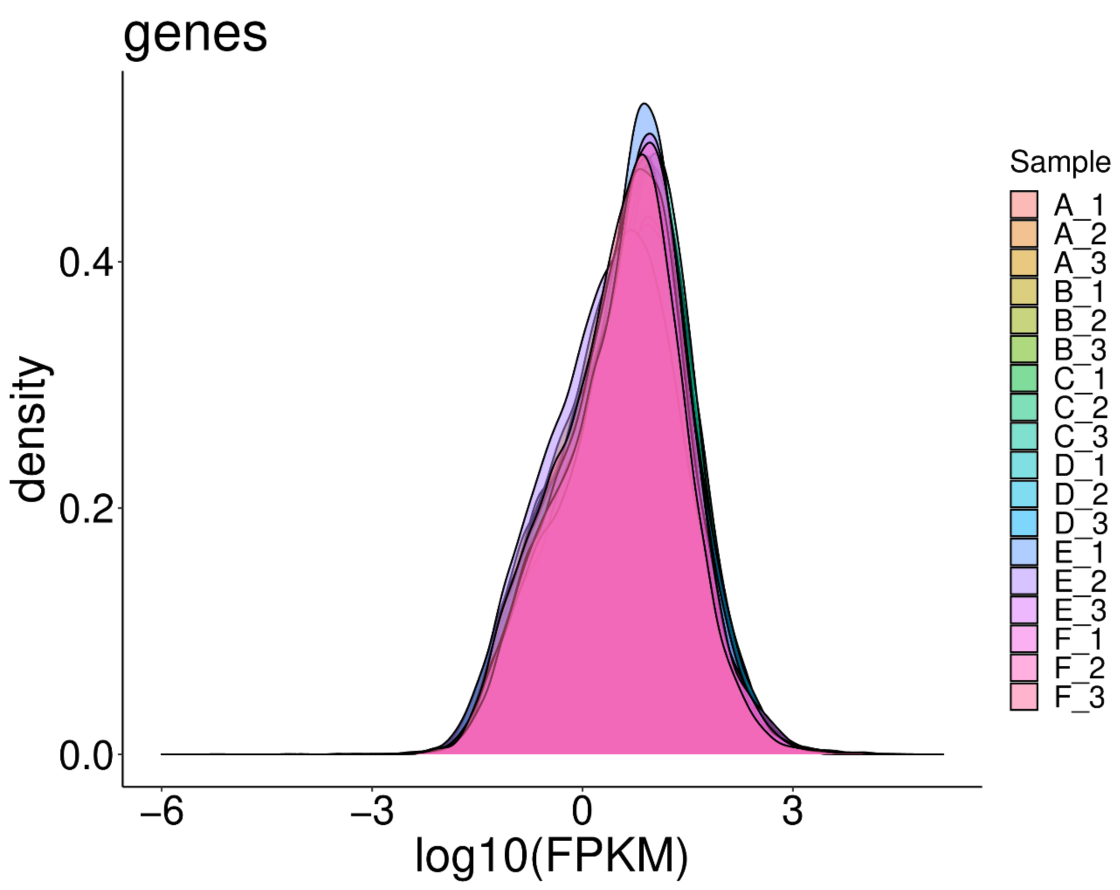
genes
density
0.4
0.2
0.0
−6
−3
0
3
log10(FPKM)
Sample
A_1
A_2
A_3
B_1
B_2
B_3
C_1
C_2
C_3
D_1
D_2
D_3
E_1
E_2
E_3
F_1
F_2
F_3

b.

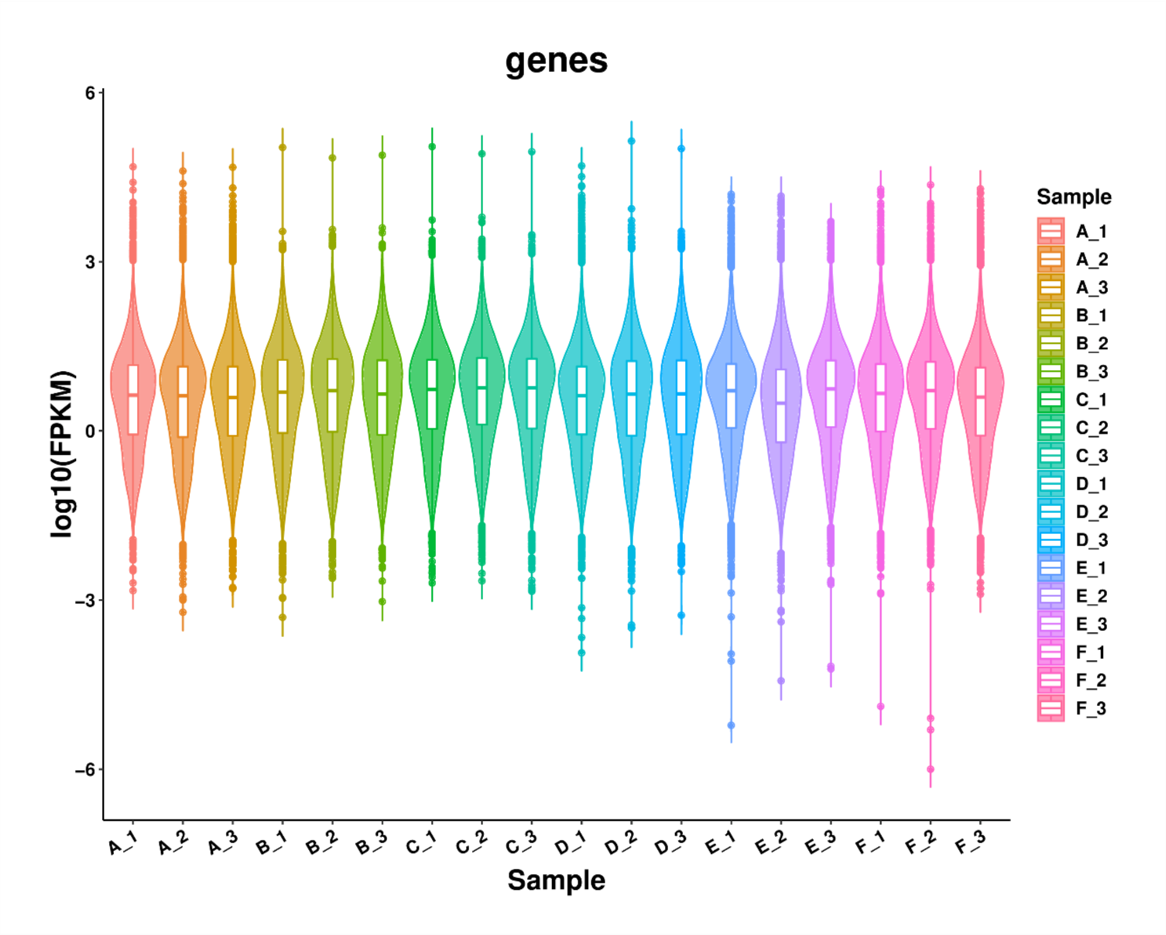
genes
log10(FPKM)
6
3
0
-3
-6
A_1
A_2
A_3
B_1
B_2
B_3
C_1
C_2
C_3
D_1
D_2
D_3
E_1
E_2
E_3
F_1
F_2
F_3
Sample
Sample
A_1
A_2
A_3
B_1
B_2
B_3
C_1
C_2
C_3
D_1
D_2
D_3
E_1
E_2
E_3
F_1
F_2
F_3

c.

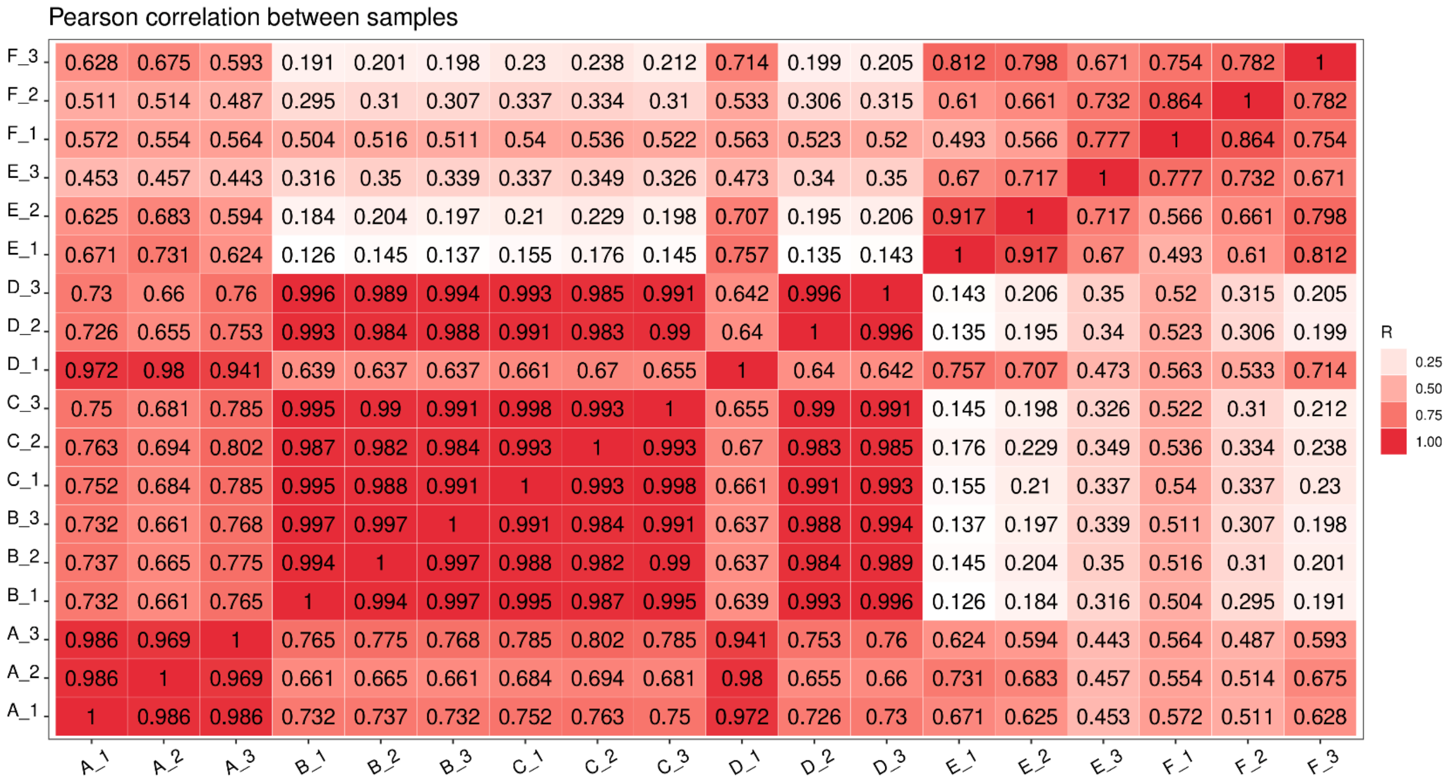

d.

Supplemental Figure S2. Gene expression statistics of all 18 samples. a. The boxplot of gene expressions in 18 samples; b. The density of gene expressions in 18 samples; c. The violin plot of gene expressions in 18 samples; d. The pearson correlation analysis in 18 samples.

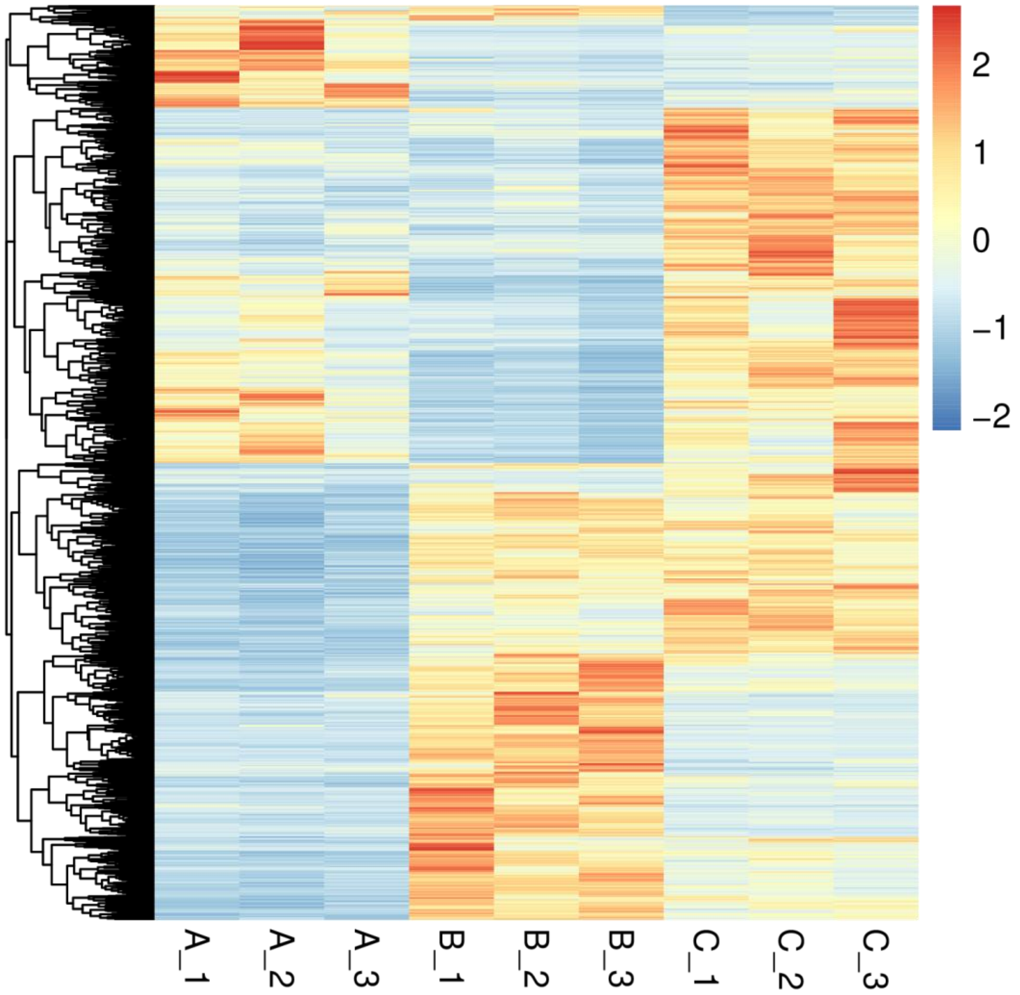
2
1
0
−1
−2
A_1
A_2
A_3
B_1
B_2
B_3
C_1
C_2
C_3

a.

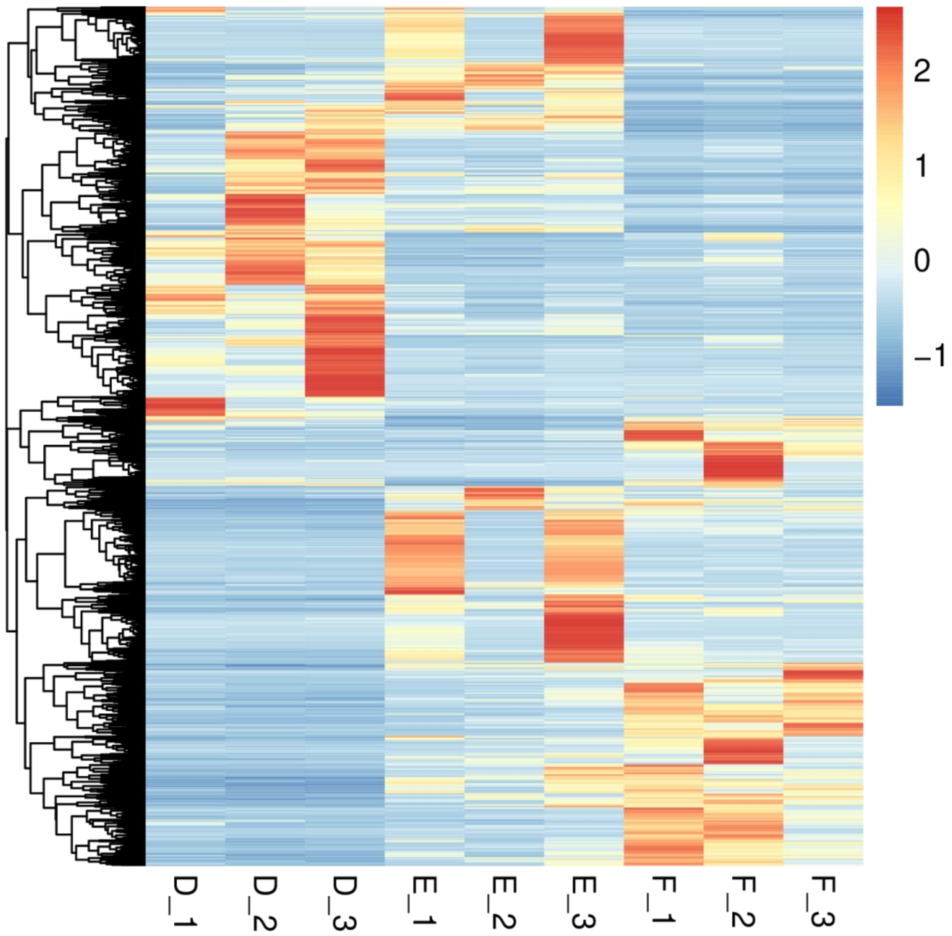

b.

Supplemental Figure S3. The gene expression heatmap of *Kingdonia uniflora* leaf tissue A vs. B vs. C (a) and flower bud tissue D vs. E vs. F (b).

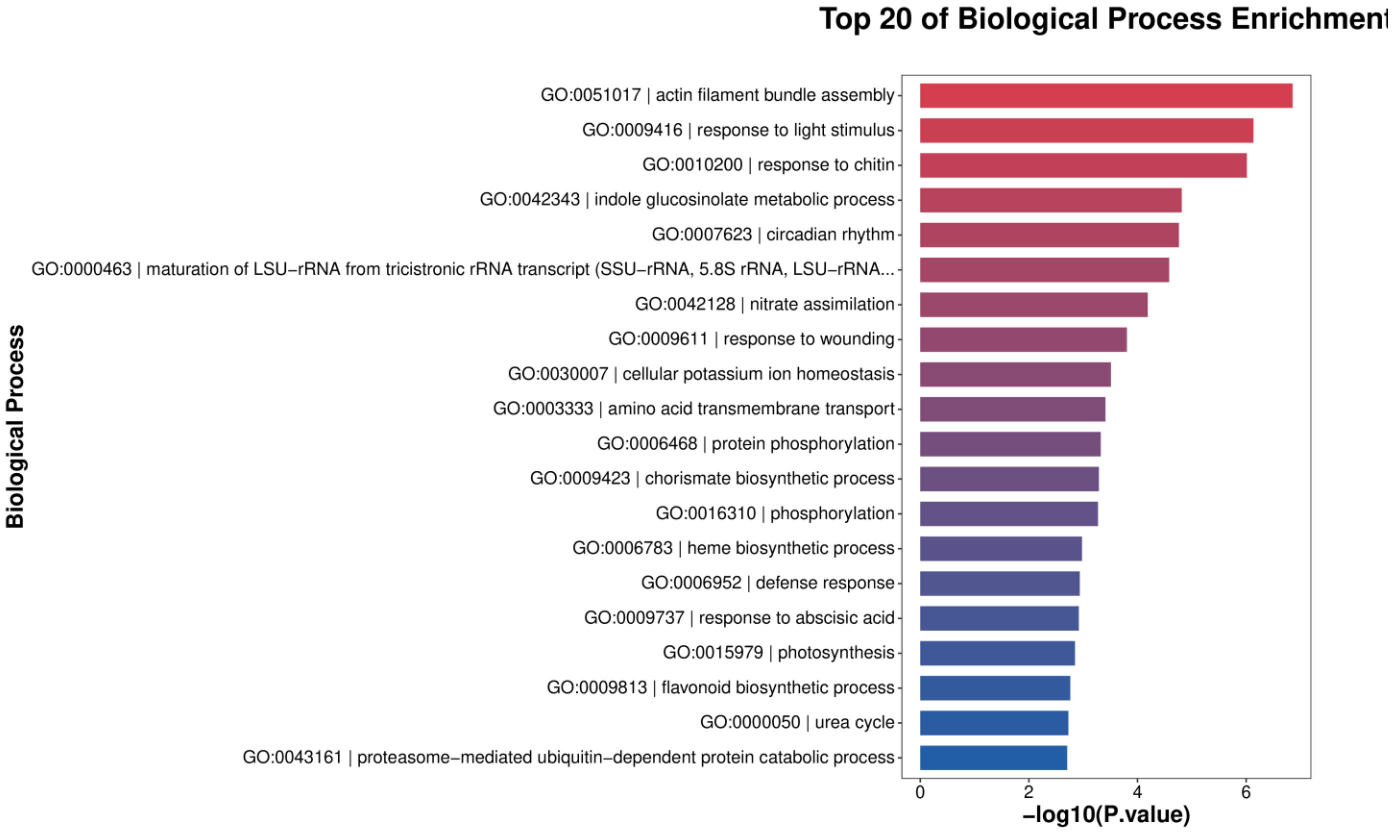
Top 20 of Biological Process Enrichment
Biological Process
GO:0051017 | actin filament bundle assembly
GO:0009416 | response to light stimulus
GO:0010200 | response to chitin
GO:0042343 | indole glucosinolate metabolic process
GO:0007623 | circadian rhythm
GO:0000463 | maturation of LSU−rRNA from tricistronic rRNA transcript (SSU−rRNA, 5.8S rRNA, LSU−rRNA...
GO:0042128 | nitrate assimilation
GO:0009611 | response to wounding
GO:0030007 | cellular potassium ion homeostasis
GO:0003333 | amino acid transmembrane transport
GO:0006468 | protein phosphorylation
GO:0009423 | chorismate biosynthetic process
GO:0016310 | phosphorylation
GO:0006783 | heme biosynthetic process
GO:0006952 | defense response
GO:0009737 | response to abscisic acid
GO:0015979 | photosynthesis
GO:0009813 | flavonoid biosynthetic process
GO:0000050 | urea cycle
GO:0043161 | proteasome−mediated ubiquitin−dependent protein catabolic process
0
2
4
6
−log10(P.value)

a.

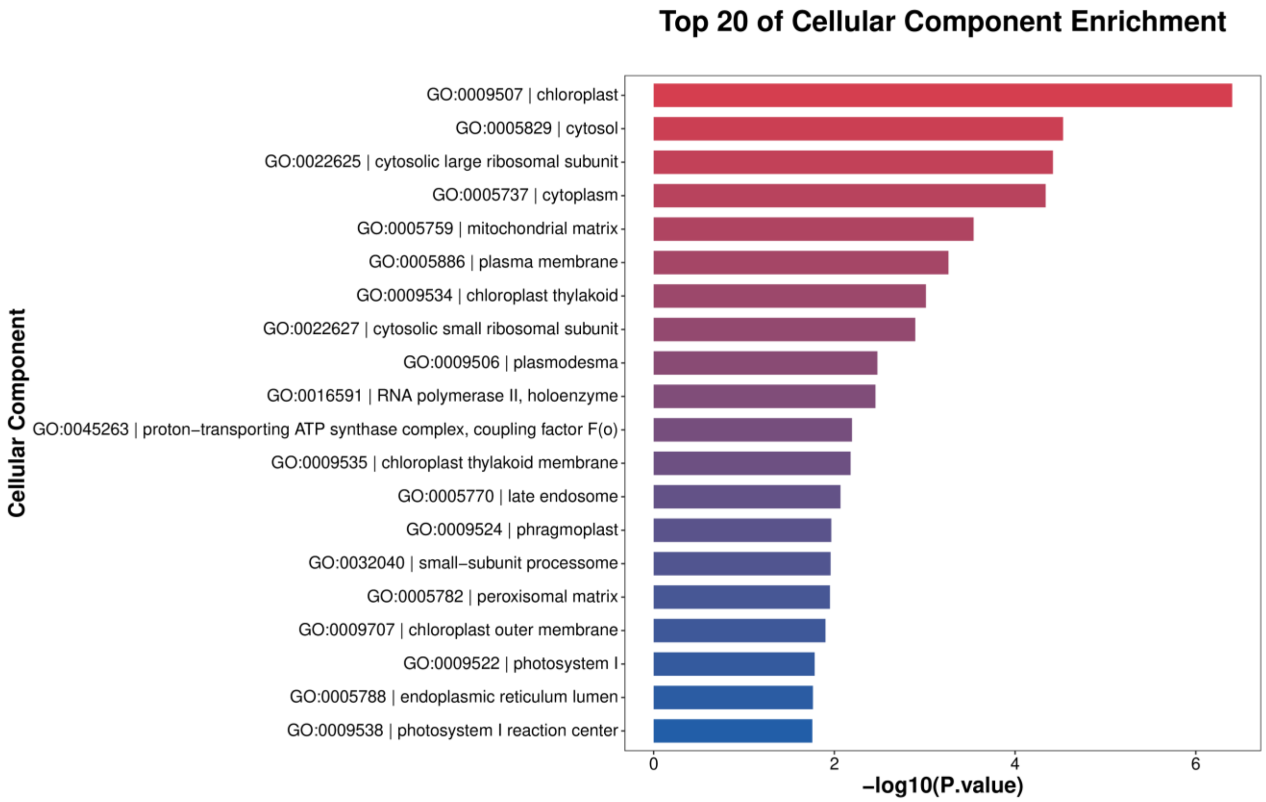
Top 20 of Cellular Component Enrichment
Cellular Component
GO:0009507 | chloroplast
GO:0005829 | cytosol
GO:0022625 | cytosolic large ribosomal subunit
GO:0005737 | cytoplasm
GO:0005759 | mitochondrial matrix
GO:0005886 | plasma membrane
GO:0009534 | chloroplast thylakoid
GO:0022627 | cytosolic small ribosomal subunit
GO:0009506 | plasmodesma
GO:0016591 | RNA polymerase II, holoenzyme
GO:0045263 | proton−transporting ATP synthase complex, coupling factor F(o)
GO:0009535 | chloroplast thylakoid membrane
GO:0005770 | late endosome
GO:0009524 | phragmoplast
GO:0032040 | small−subunit processome
GO:0005782 | peroxisomal matrix
GO:0009707 | chloroplast outer membrane
GO:0009522 | photosystem I
GO:0005788 | endoplasmic reticulum lumen
GO:0009538 | photosystem I reaction center
0
2
4
6
−log10(P.value)

b.

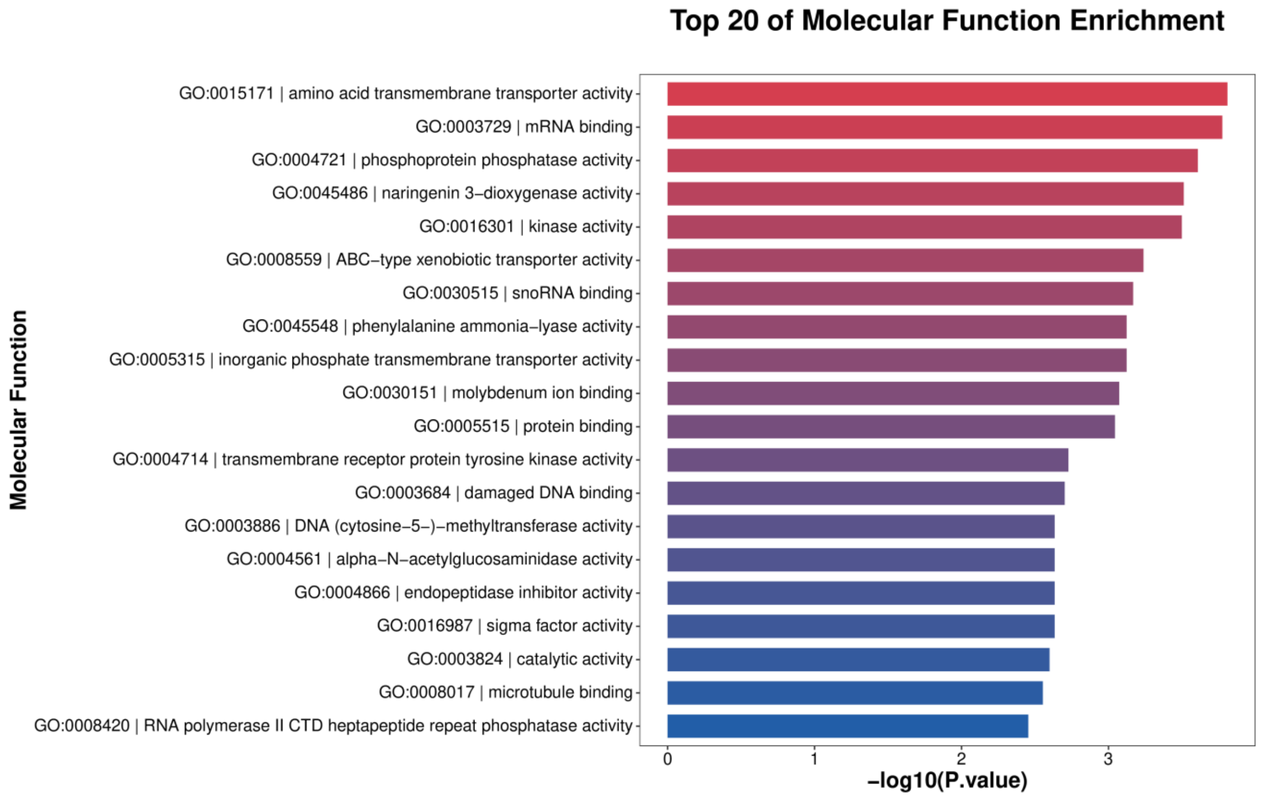

c.

Supplemental Figure S4. The GO enrichment results of DEGs in *Kingdonia uniflora* leaf tissue A vs. B vs. C. a. Biological Process ; b. Cellular Component; c. Molecular Function.

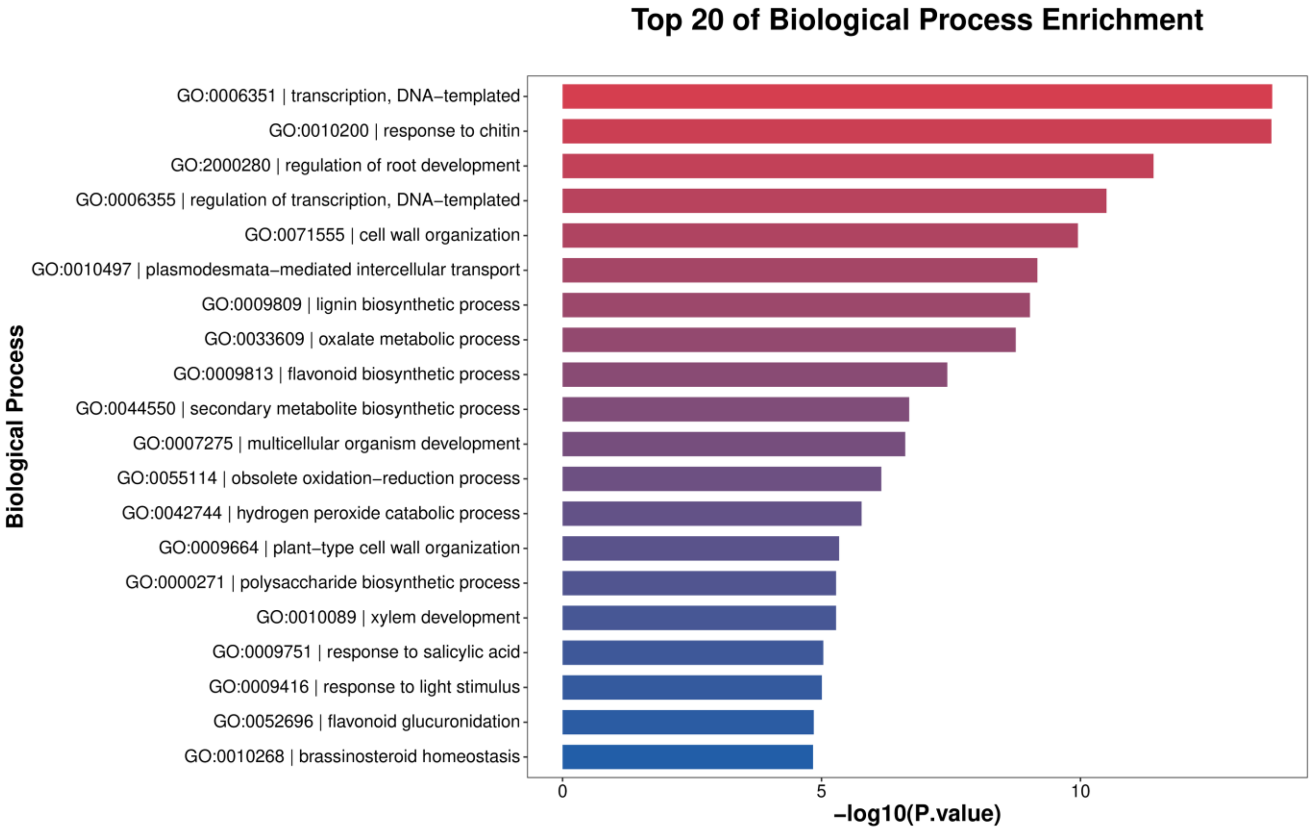
Top 20 of Biological Process Enrichment
Biological Process
GO:0006351 | transcription, DNA−templated
GO:0010200 | response to chitin
GO:2000280 | regulation of root development
GO:0006355 | regulation of transcription, DNA−templated
GO:0071555 | cell wall organization
GO:0010497 | plasmodesmata−mediated intercellular transport
GO:0009809 | lignin biosynthetic process
GO:0033609 | oxalate metabolic process
GO:0009813 | flavonoid biosynthetic process
GO:0044550 | secondary metabolite biosynthetic process
GO:0007275 | multicellular organism development
GO:0055114 | obsolete oxidation−reduction process
GO:0042744 | hydrogen peroxide catabolic process
GO:0009664 | plant−type cell wall organization
GO:0000271 | polysaccharide biosynthetic process
GO:0010089 | xylem development
GO:0009751 | response to salicylic acid
GO:0009416 | response to light stimulus
GO:0052696 | flavonoid glucuronidation
GO:0010268 | brassinosteroid homeostasis
0
5
10
−log10(P.value)

a.

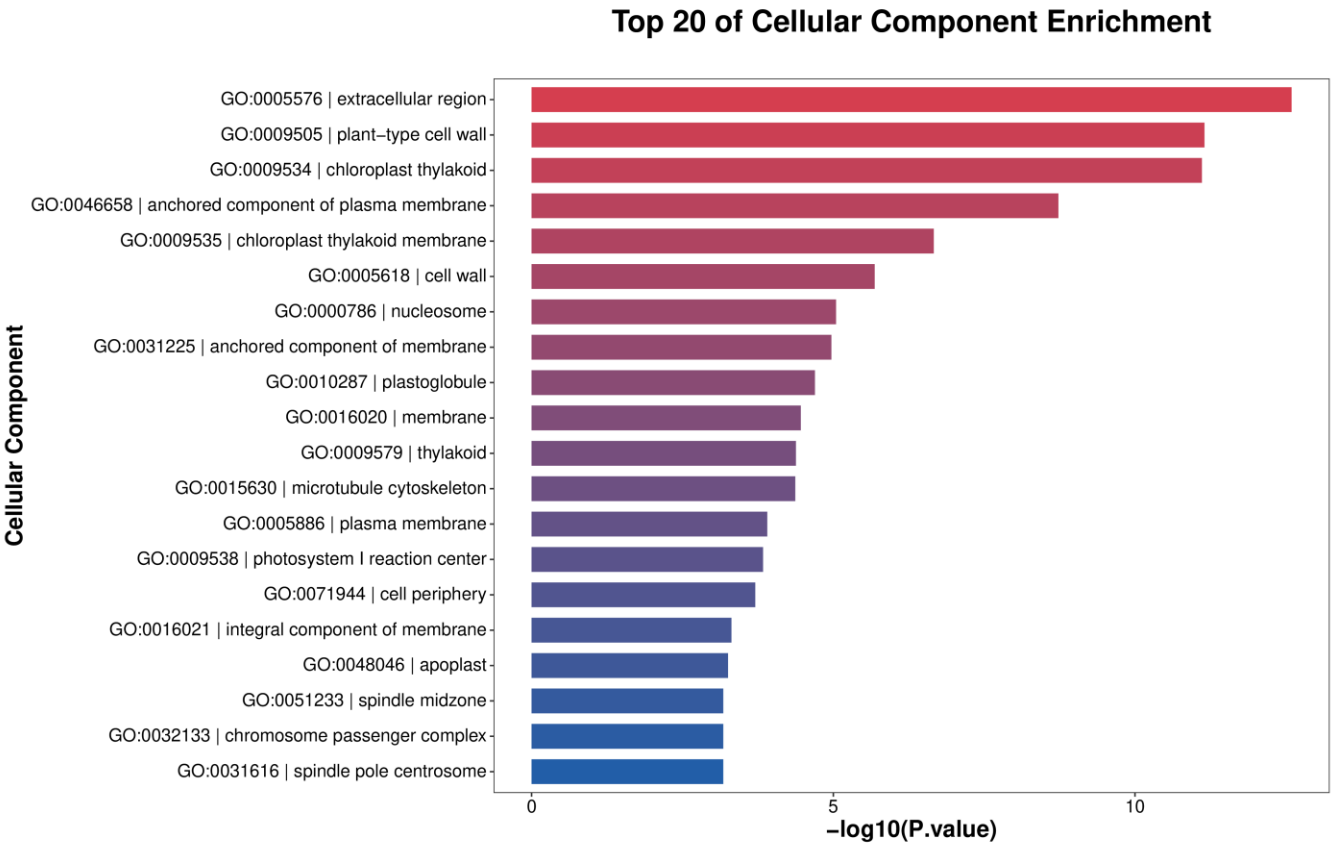
Top 20 of Cellular Component Enrichment
Cellular Component
GO:0005576 | extracellular region
GO:0009505 | plant−type cell wall
GO:0009534 | chloroplast thylakoid
GO:0046658 | anchored component of plasma membrane
GO:0009535 | chloroplast thylakoid membrane
GO:0005618 | cell wall
GO:0000786 | nucleosome
GO:0031225 | anchored component of membrane
GO:0010287 | plastoglobule
GO:0016020 | membrane
GO:0009579 | thylakoid
GO:0015630 | microtubule cytoskeleton
GO:0005886 | plasma membrane
GO:0009538 | photosystem I reaction center
GO:0071944 | cell periphery
GO:0016021 | integral component of membrane
GO:0048046 | apoplast
GO:0051233 | spindle midzone
GO:0032133 | chromosome passenger complex
GO:0031616 | spindle pole centrosome
0
5
10
−log10(P.value)

b.

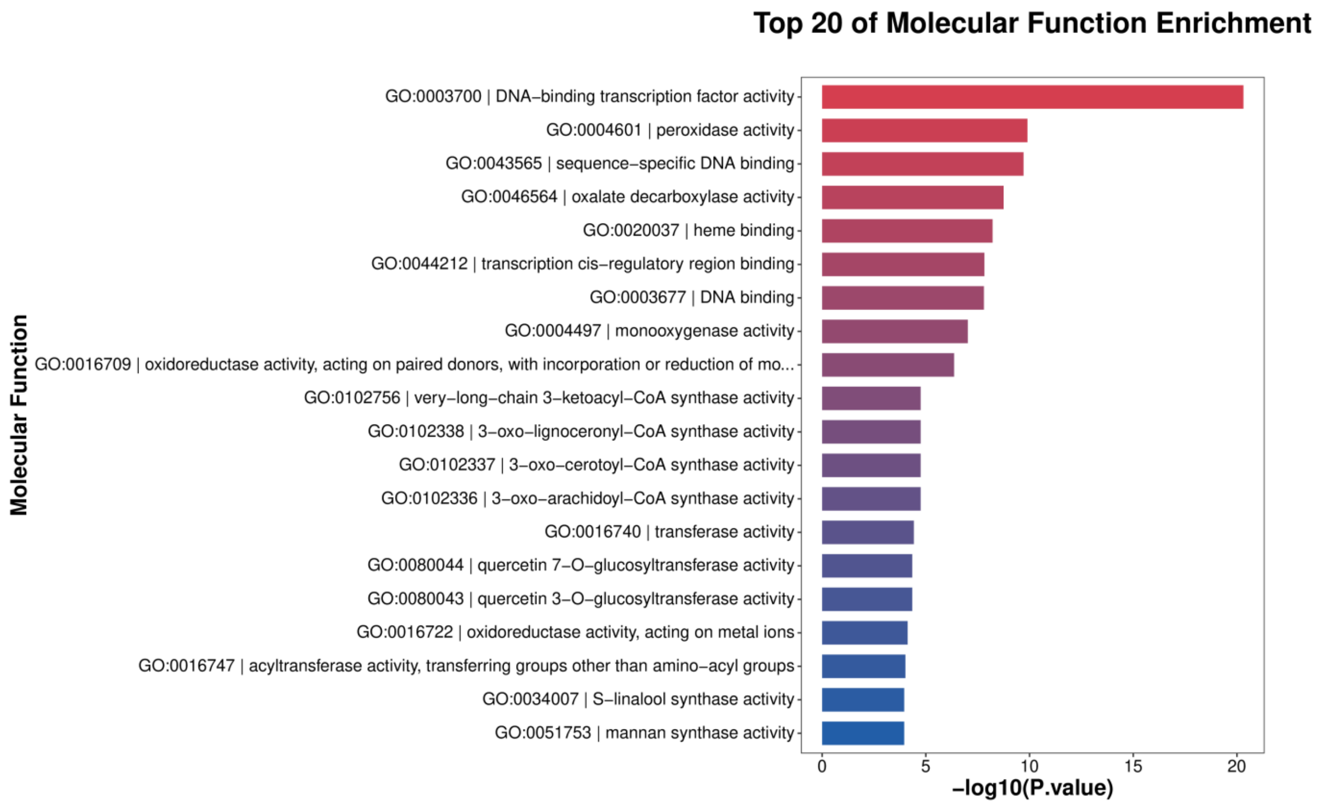

c.

Supplemental Figure S5. The GO enrichment results of DEGs in *Kingdonia uniflora* flower bud tissue D vs. E vs. F. a. Biological Process ; b. Cellular Component; c. Molecular Function.

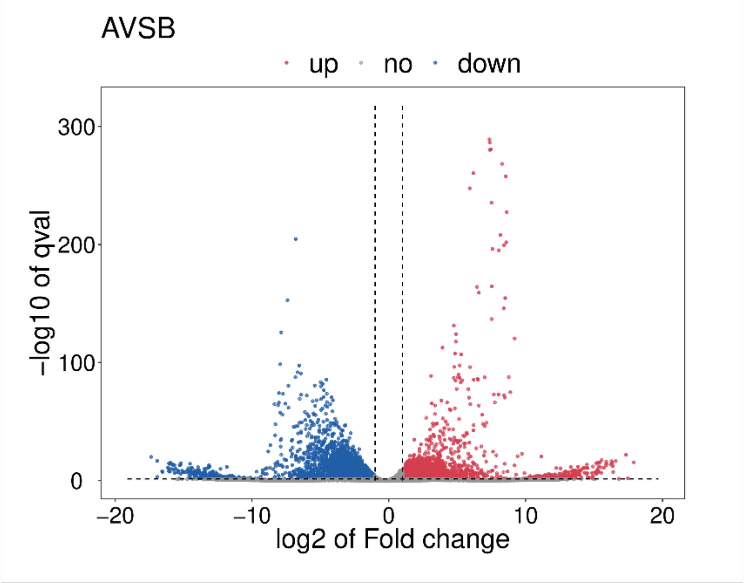
AVSB
up
no
down
300
200
100
0
−log10 of qval
−20
−10
0
10
20
log2 of Fold change

a.

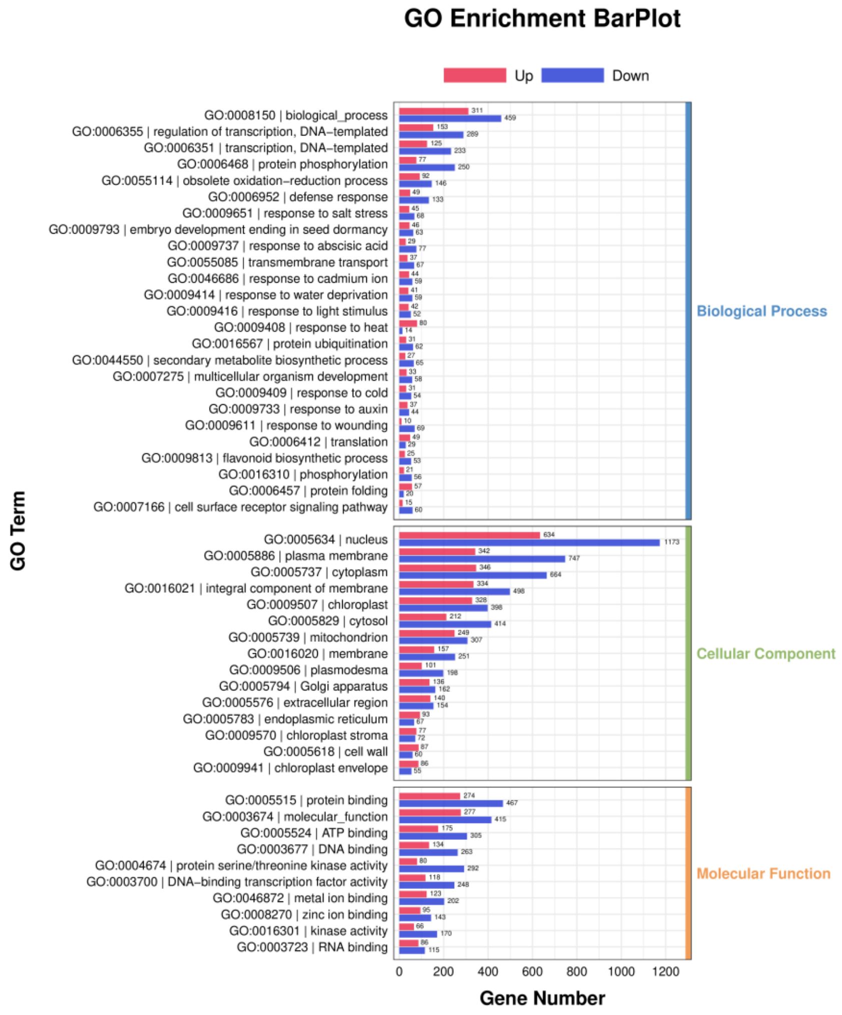
GO Enrichment BarPlot
Up
Down
GO:0008150 | biological_process
GO:0006355 | regulation of transcription, DNA−templated
GO:0006351 | transcription, DNA−templated
GO:0006468 | protein phosphorylation
GO:0055114 | obsolete oxidation−reduction process
GO:0006952 | defense response
GO:0009651 | response to salt stress
GO:0009793 | embryo development ending in seed dormancy
GO:0009737 | response to abscisic acid
GO:0055085 | transmembrane transport
GO:0046686 | response to cadmium ion
GO:0009414 | response to water deprivation
GO:0009416 | response to light stimulus
GO:0009408 | response to heat
GO:0016567 | protein ubiquitination
GO:0044550 | secondary metabolite biosynthetic process
GO:0007275 | multicellular organism development
GO:0009409 | response to cold
GO:0009733 | response to auxin
GO:0009611 | response to wounding
GO:0006412 | translation
GO:0009813 | flavonoid biosynthetic process
GO:0016310 | phosphorylation
GO:0006457 | protein folding
GO:0007166 | cell surface receptor signaling pathway
GO:0005634 | nucleus
GO:0005886 | plasma membrane
GO:0005737 | cytoplasm
GO:0016021 | integral component of membrane
GO:0009507 | chloroplast
GO:0005829 | cytosol
GO:0005739 | mitochondrion
GO:0016020 | membrane
GO:0009506 | plasmodesma
GO:0005794 | Golgi apparatus
GO:0005576 | extracellular region
GO:0005783 | endoplasmic reticulum
GO:0009570 | chloroplast stroma
GO:0005618 | cell wall
GO:0009941 | chloroplast envelope
GO:0005515 | protein binding
GO:0003674 | molecular_function
GO:0005524 | ATP binding
GO:0003677 | DNA binding
GO:0004674 | protein serine/threonine kinase activity
GO:0003700 | DNA−binding transcription factor activity
GO:0046872 | metal ion binding
GO:0008270 | zinc ion binding
GO:0016301 | kinase activity
GO:0003723 | RNA binding
GO Term
Biological Process
Cellular Component
Molecular Function
0
200
400
600
800
1000
1200
Gene Number

b.

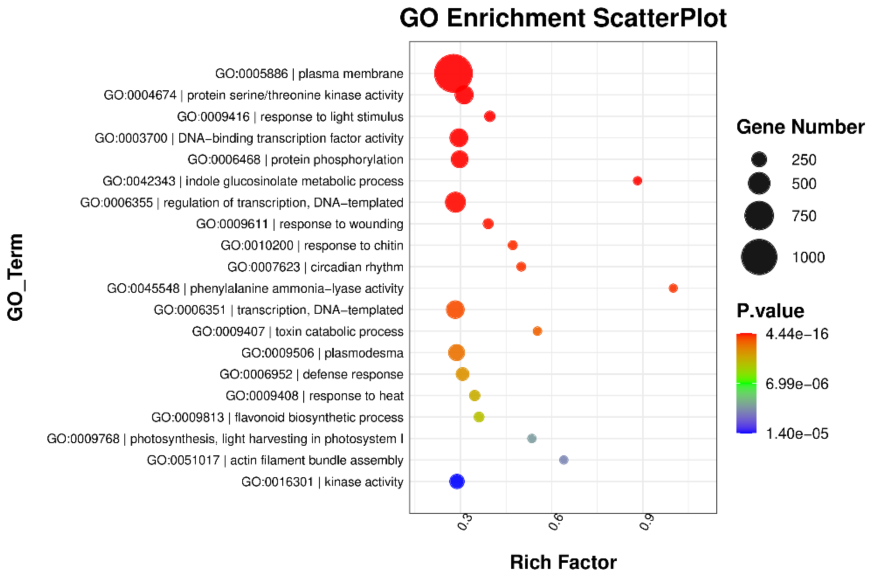
GO Enrichment ScatterPlot
GO:0005886 | plasma membrane
GO:0004674 | protein serine/threonine kinase activity
GO:0009416 | response to light stimulus
GO:0003700 | DNA–binding transcription factor activity
GO:0006468 | protein phosphorylation
GO:0042343 | indole glucosinolate metabolic process
GO:0006355 | regulation of transcription, DNA–templated
GO:0009611 | response to wounding
GO:0010200 | response to chitin
GO:0007623 | circadian rhythm
GO:0045548 | phenylalanine ammonia–lyase activity
GO:0006351 | transcription, DNA–templated
GO:0009407 | toxin catabolic process
GO:0009506 | plasmodesma
GO:0006952 | defense response
GO:0009408 | response to heat
GO:0009813 | flavonoid biosynthetic process
GO:0009768 | photosynthesis, light harvesting in photosystem I
GO:0051017 | actin filament bundle assembly
GO:0016301 | kinase activity
GO_Term
0.3
0.6
0.9
Rich Factor
Gene Number
250
500
750
1000
P.value
4.44e−16
6.99e−06
1.40e−05
c.

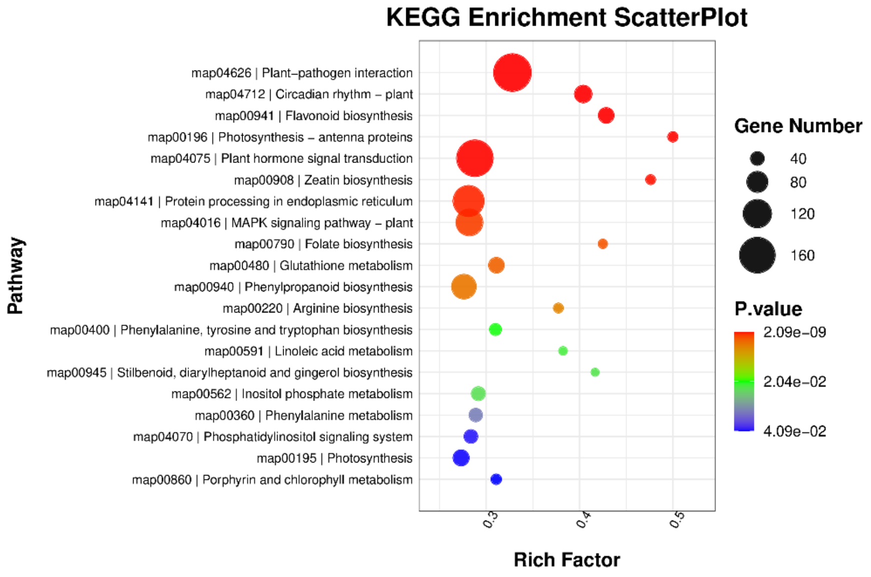
KEGG Enrichment ScatterPlot
map04626 | Plant–pathogen interaction
map04712 | Circadian rhythm – plant
map00941 | Flavonoid biosynthesis
map00196 | Photosynthesis – antenna proteins
map04075 | Plant hormone signal transduction
map00908 | Zeatin biosynthesis
map04141 | Protein processing in endoplasmic reticulum
map04016 | MAPK signaling pathway – plant
map00790 | Folate biosynthesis
map00480 | Glutathione metabolism
map00940 | Phenylpropanoid biosynthesis
map00220 | Arginine biosynthesis
map00400 | Phenylalanine, tyrosine and tryptophan biosynthesis
map00591 | Linoleic acid metabolism
map00945 | Stilbenoid, diarylheptanoid and gingerol biosynthesis
map00562 | Inositol phosphate metabolism
map00360 | Phenylalanine metabolism
map04070 | Phosphatidylinositol signaling system
map00195 | Photosynthesis
map00860 | Porphyrin and chlorophyll metabolism
Pathway
0.3
0.4
0.5
Rich Factor
Gene Number
40
80
120
160
P.value
2.09e−09
2.04e−02
4.09e−02
d.

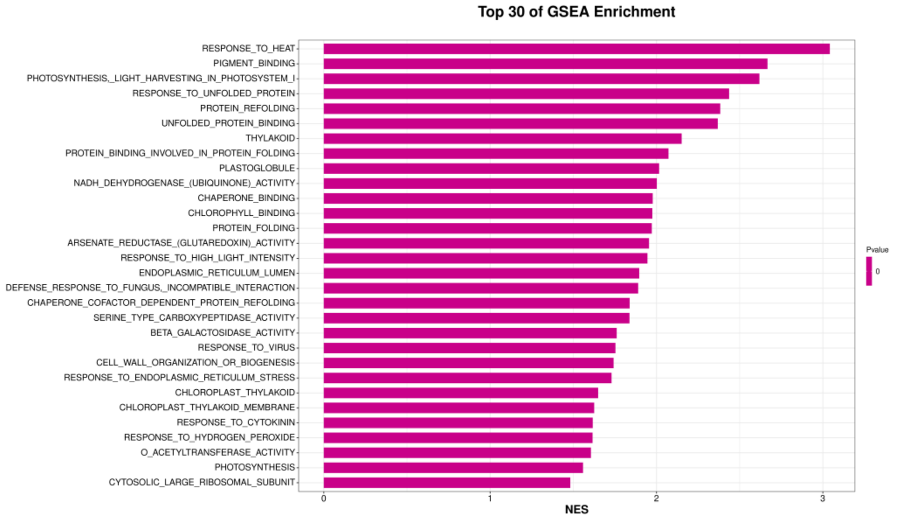

e.

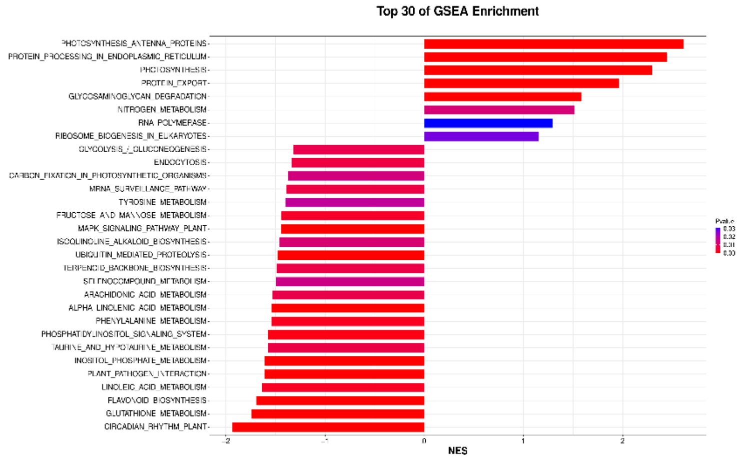

f.

Supplemental Figure 6. The volcano plot of gene expression pattern (a), the GO enrichment bar plot (b), the GO enrichment scatter plot (c), the KEGG enrichment scatter plot (d), the GO Gsea enrichment analysis (e) and the KEGG Gsea enrichment analysis (f) of DEGs between leaf tissue A and B group.

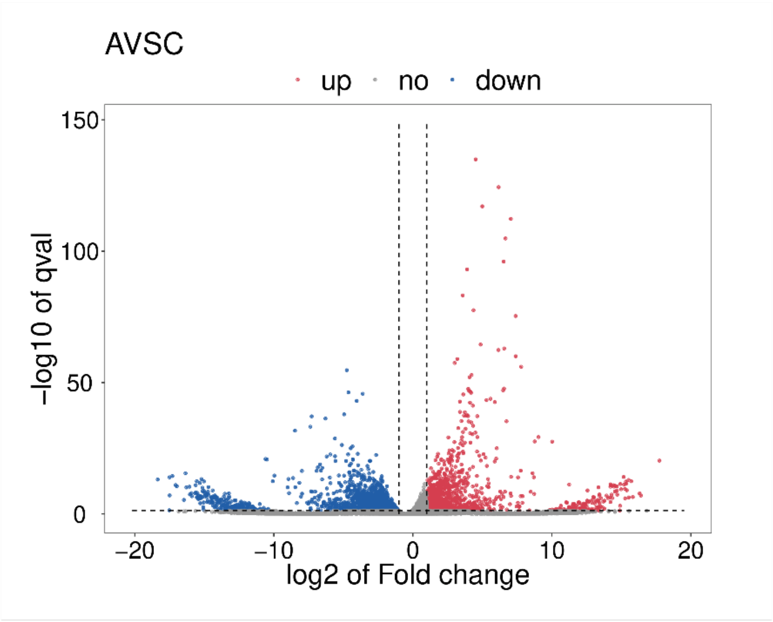
AVSC
up
no
down
150
100
50
0
−log10 of qval
−20
−10
0
10
20
log2 of Fold change

a.

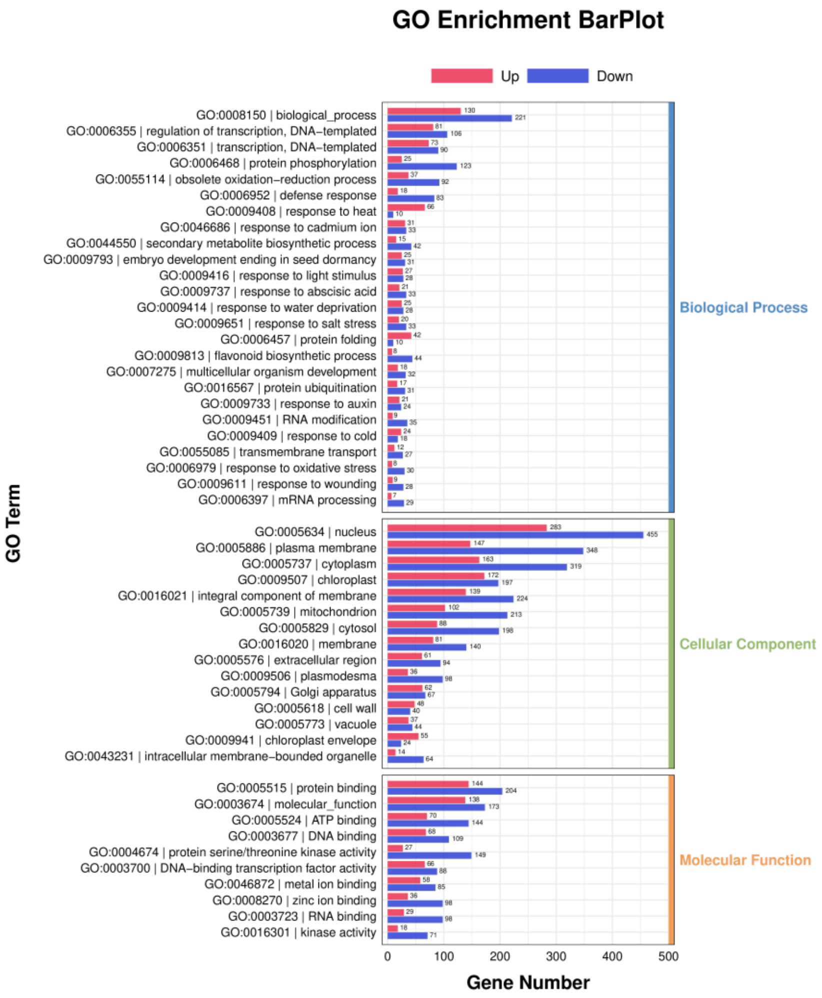
GO Enrichment BarPlot
Up
Down
GO:0008150 | biological_process
GO:0006355 | regulation of transcription, DNA−templated
GO:0006351 | transcription, DNA−templated
GO:0006468 | protein phosphorylation
GO:0055114 | obsolete oxidation−reduction process
GO:0006952 | defense response
GO:0009408 | response to heat
GO:0046686 | response to cadmium ion
GO:0044550 | secondary metabolite biosynthetic process
GO:0009793 | embryo development ending in seed dormancy
GO:0009416 | response to light stimulus
GO:0009737 | response to abscisic acid
GO:0009414 | response to water deprivation
GO:0009651 | response to salt stress
GO:0006457 | protein folding
GO:0009813 | flavonoid biosynthetic process
GO:0007275 | multicellular organism development
GO:0016567 | protein ubiquitination
GO:0009733 | response to auxin
GO:0009451 | RNA modification
GO:0009409 | response to cold
GO:0055085 | transmembrane transport
GO:0006979 | response to oxidative stress
GO:0009611 | response to wounding
GO:0006397 | mRNA processing
GO:0005634 | nucleus
GO:0005886 | plasma membrane
GO:0005737 | cytoplasm
GO:0009507 | chloroplast
GO:0016021 | integral component of membrane
GO:0005739 | mitochondrion
GO:0005829 | cytosol
GO:0016020 | membrane
GO:0005576 | extracellular region
GO:0009506 | plasmodesma
GO:0005794 | Golgi apparatus
GO:0005618 | cell wall
GO:0005773 | vacuole
GO:0009941 | chloroplast envelope
GO:0043231 | intracellular membrane−bounded organelle
GO:0005515 | protein binding
GO:0003674 | molecular_function
GO:0005524 | ATP binding
GO:0003677 | DNA binding
GO:0004674 | protein serine/threonine kinase activity
GO:0003700 | DNA−binding transcription factor activity
GO:0046872 | metal ion binding
GO:0008270 | zinc ion binding
GO:0003723 | RNA binding
GO:0016301 | kinase activity
GO Term
Biological Process
Cellular Component
Molecular Function
0
100
200
300
400
500
Gene Number

b.

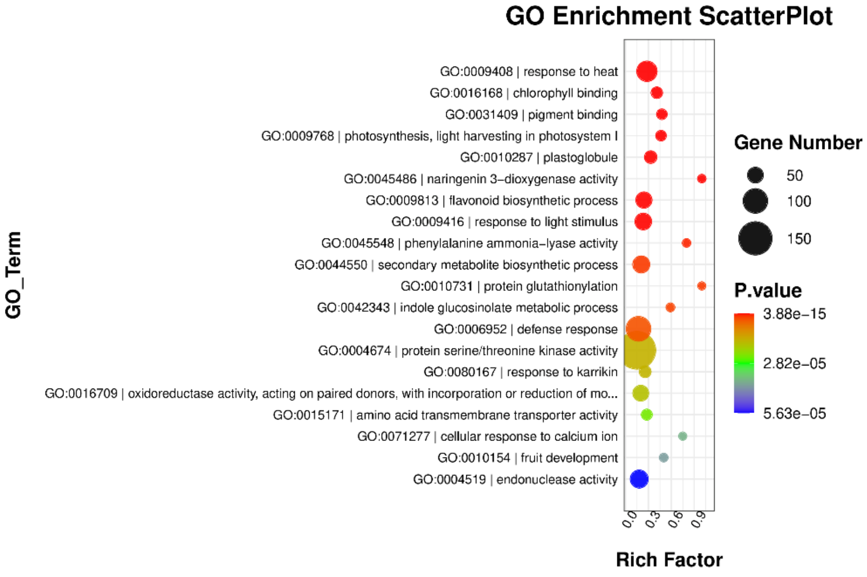
GO Enrichment ScatterPlot
GO:0009408 | response to heat
GO:0016168 | chlorophyll binding
GO:0031409 | pigment binding
GO:0009768 | photosynthesis, light harvesting in photosystem I
GO:0010287 | plastoglobule
GO:0045486 | naringenin 3–dioxygenase activity
GO:0009813 | flavonoid biosynthetic process
GO:0009416 | response to light stimulus
GO:0045548 | phenylalanine ammonia–lyase activity
GO:0044550 | secondary metabolite biosynthetic process
GO:0010731 | protein glutathionylation
GO:0042343 | indole glucosinolate metabolic process
GO:0006952 | defense response
GO:0004674 | protein serine/threonine kinase activity
GO:0080167 | response to karrikin
GO:0016709 | oxidoreductase activity, acting on paired donors, with incorporation or reduction of mo...
GO:0015171 | amino acid transmembrane transporter activity
GO:0071277 | cellular response to calcium ion
GO:0010154 | fruit development
GO:0004519 | endonuclease activity
GO_Term
0.0
0.3
0.6
0.9
Rich Factor
Gene Number
50
100
150
P.value
3.88e−15
2.82e−05
5.63e−05

c.

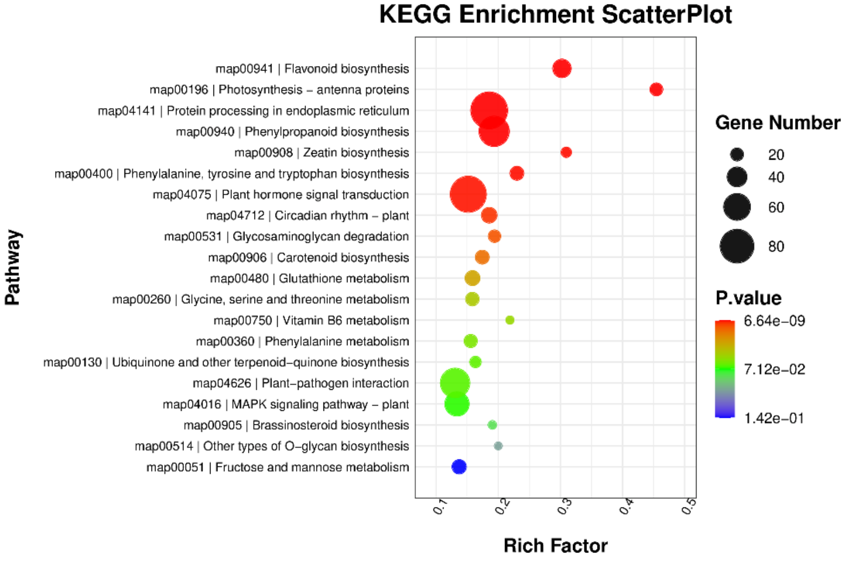
KEGG Enrichment ScatterPlot
map00941 | Flavonoid biosynthesis
map00196 | Photosynthesis – antenna proteins
map04141 | Protein processing in endoplasmic reticulum
map00940 | Phenylpropanoid biosynthesis
map00908 | Zeatin biosynthesis
map00400 | Phenylalanine, tyrosine and tryptophan biosynthesis
map04075 | Plant hormone signal transduction
map04712 | Circadian rhythm – plant
map00531 | Glycosaminoglycan degradation
map00906 | Carotenoid biosynthesis
map00480 | Glutathione metabolism
map00260 | Glycine, serine and threonine metabolism
map00750 | Vitamin B6 metabolism
map00360 | Phenylalanine metabolism
map00130 | Ubiquinone and other terpenoid–quinone biosynthesis
map04626 | Plant–pathogen interaction
map04016 | MAPK signaling pathway – plant
map00905 | Brassinosteroid biosynthesis
map00514 | Other types of O–glycan biosynthesis
map00051 | Fructose and mannose metabolism
Pathway
0.1
0.2
0.3
0.4
0.5
Rich Factor
Gene Number
20
40
60
80
P.value
6.64e−09
7.12e−02
1.42e−01

d.

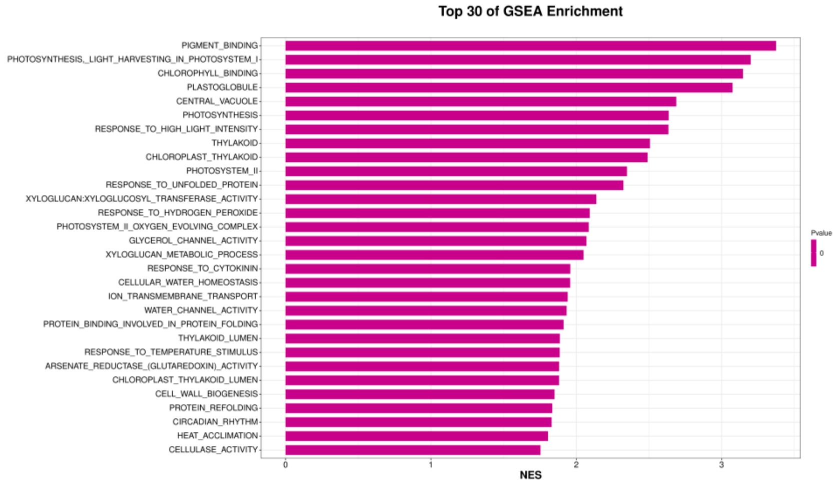

e.

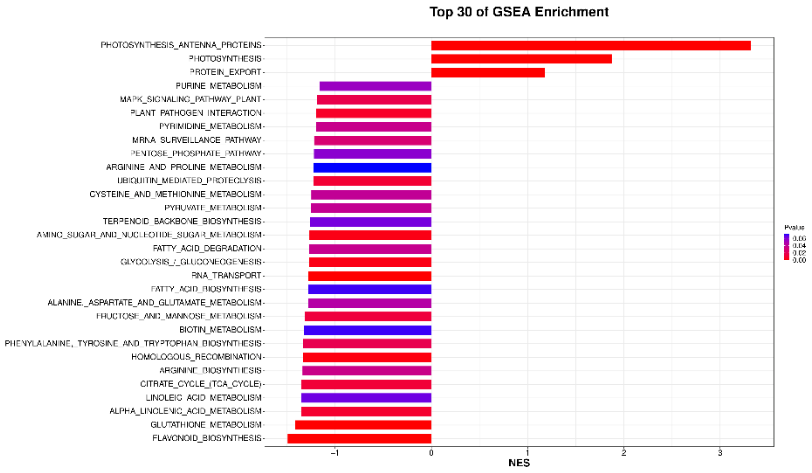

f.

Supplemental Figure S7. The volcano plot of gene expression pattern (a), the GO enrichment bar plot (b), the GO enrichment scatter plot (c), the KEGG enrichment scatter plot (d), the GO Gsea enrichment analysis (e) and the KEGG Gsea enrichment analysis (f) of DEGs between leaf tissue A and C group.

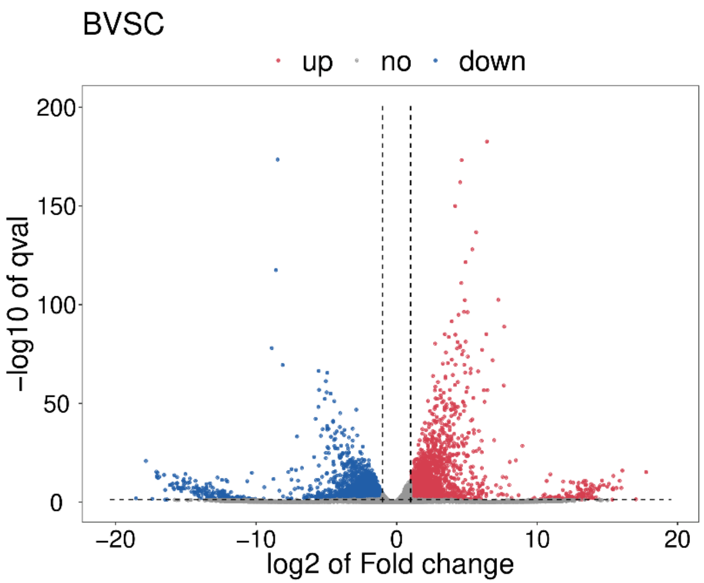
BVSC
up
no
down
200
150
100
50
0
−log10 of qval
−20
−10
0
10
20
log2 of Fold change

a.

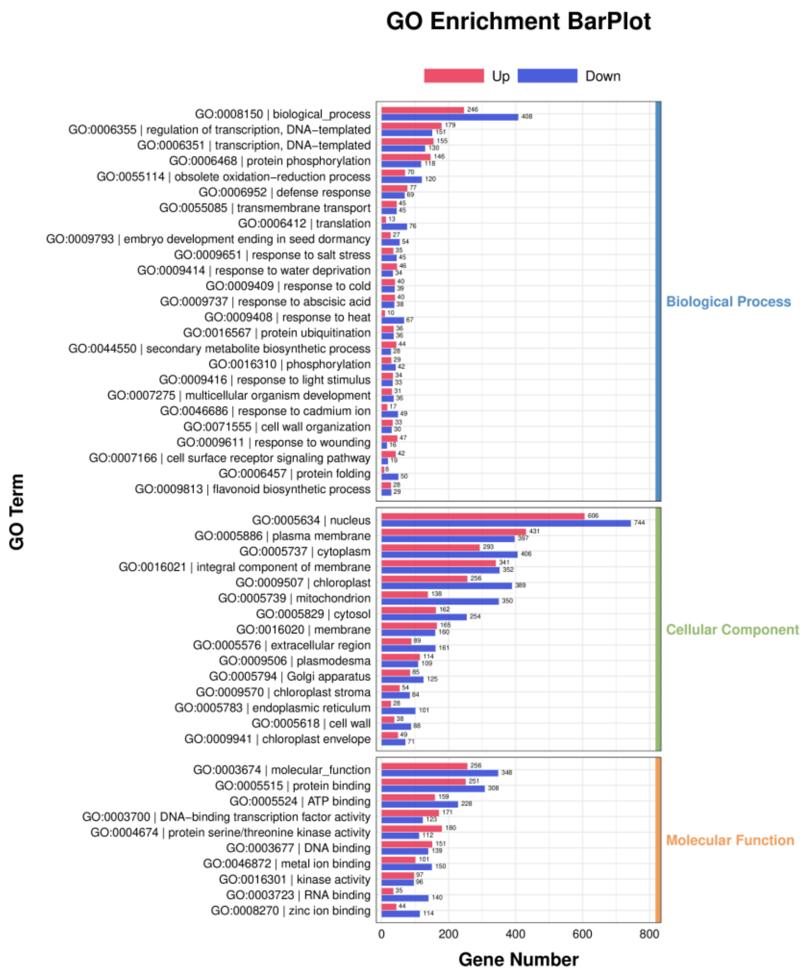
GO Enrichment BarPlot
Up
Down
GO:0008150 | biological_process
GO:0006355 | regulation of transcription, DNA−templated
GO:0006351 | transcription, DNA−templated
GO:0006468 | protein phosphorylation
GO:0055114 | obsolete oxidation−reduction process
GO:0006952 | defense response
GO:0055085 | transmembrane transport
GO:0006412 | translation
GO:0009793 | embryo development ending in seed dormancy
GO:0009651 | response to salt stress
GO:0009414 | response to water deprivation
GO:0009409 | response to cold
GO:0009737 | response to abscisic acid
GO:0009408 | response to heat
GO:0016567 | protein ubiquitination
GO:0044550 | secondary metabolite biosynthetic process
GO:0016310 | phosphorylation
GO:0009416 | response to light stimulus
GO:0007275 | multicellular organism development
GO:0046686 | response to cadmium ion
GO:0071555 | cell wall organization
GO:0009611 | response to wounding
GO:0007166 | cell surface receptor signaling pathway
GO:0006457 | protein folding
GO:0009813 | flavonoid biosynthetic process
GO:0005634 | nucleus
GO:0005886 | plasma membrane
GO:0005737 | cytoplasm
GO:0016021 | integral component of membrane
GO:0009507 | chloroplast
GO:0005739 | mitochondrion
GO:0005829 | cytosol
GO:0016020 | membrane
GO:0005576 | extracellular region
GO:0009506 | plasmodesma
GO:0005794 | Golgi apparatus
GO:0009570 | chloroplast stroma
GO:0005783 | endoplasmic reticulum
GO:0005618 | cell wall
GO:0009941 | chloroplast envelope
GO:0003674 | molecular_function
GO:0005515 | protein binding
GO:0005524 | ATP binding
GO:0003700 | DNA−binding transcription factor activity
GO:0004674 | protein serine/threonine kinase activity
GO:0003677 | DNA binding
GO:0046872 | metal ion binding
GO:0016301 | kinase activity
GO:0003723 | RNA binding
GO:0008270 | zinc ion binding
GO Term
Biological Process
Cellular Component
Molecular Function
0
200
400
600
800
Gene Number

b.

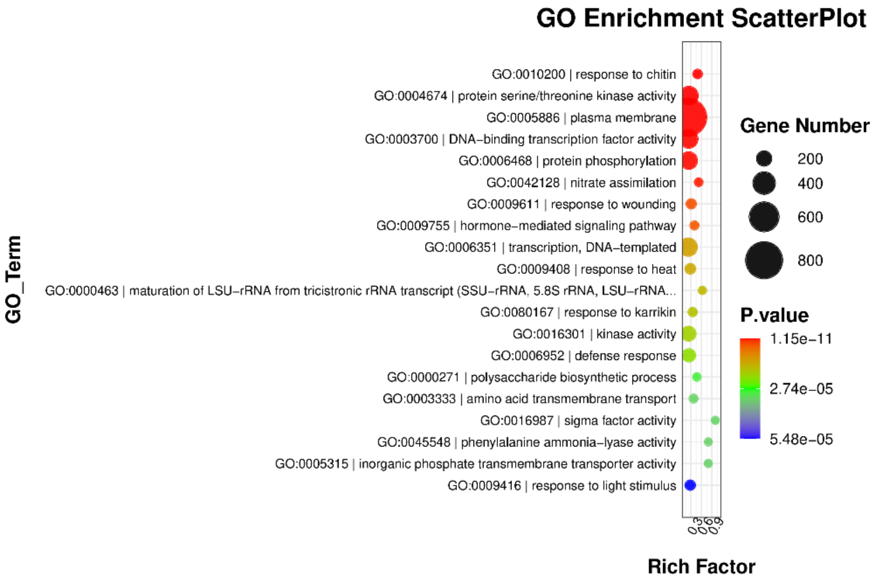

GO Enrichment ScatterPlot
GO:0010200 | response to chitin
GO:0004674 | protein serine/threonine kinase activity
GO:0005886 | plasma membrane
GO:0003700 | DNA−binding transcription factor activity
GO:0006468 | protein phosphorylation
GO:0042128 | nitrate assimilation
GO:0009611 | response to wounding
GO:0009755 | hormone−mediated signaling pathway
GO:0006351 | transcription, DNA−templated
GO:0009408 | response to heat
GO:0000463 | maturation of LSU−rRNA from tricistronic rRNA transcript (SSU−rRNA, 5.8S rRNA, LSU−rRNA...
GO:0080167 | response to karrikin
GO:0016301 | kinase activity
GO:0006952 | defense response
GO:0000271 | polysaccharide biosynthetic process
GO:0003333 | amino acid transmembrane transport
GO:0016987 | sigma factor activity
GO:0045548 | phenylalanine ammonia−lyase activity
GO:0005315 | inorganic phosphate transmembrane transporter activity
GO:0009416 | response to light stimulus
GO_Term
Rich Factor
Gene Number
200
400
600
800
P.value
1.15e−11
2.74e−05
5.48e−05

c.

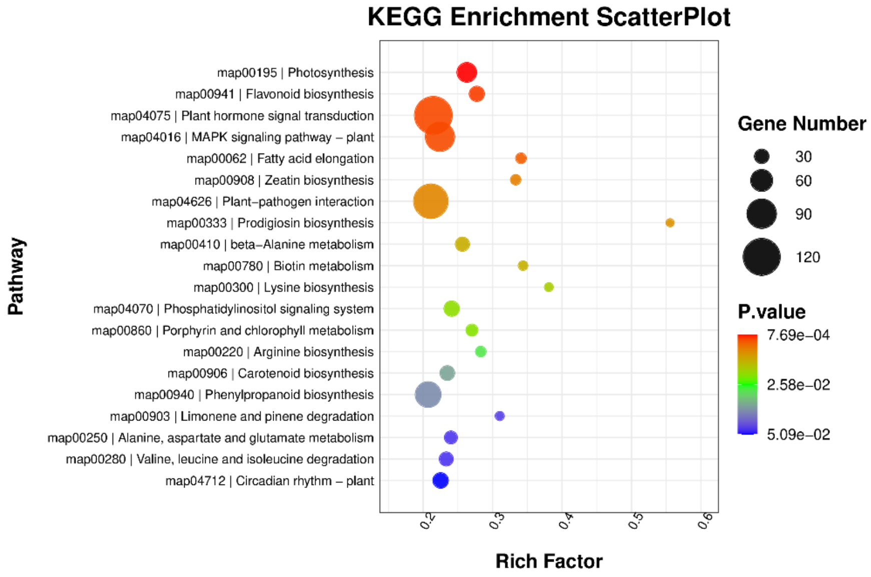

KEGG Enrichment ScatterPlot
map00195 | Photosynthesis
map00941 | Flavonoid biosynthesis
map04075 | Plant hormone signal transduction
map04016 | MAPK signaling pathway − plant
map00062 | Fatty acid elongation
map00908 | Zeatin biosynthesis
map04626 | Plant−pathogen interaction
map00333 | Prodigiosin biosynthesis
map00410 | beta−Alanine metabolism
map00780 | Biotin metabolism
map00300 | Lysine biosynthesis
map04070 | Phosphatidylinositol signaling system
map00860 | Porphyrin and chlorophyll metabolism
map00220 | Arginine biosynthesis
map00906 | Carotenoid biosynthesis
map00940 | Phenylpropanoid biosynthesis
map00903 | Limonene and pinene degradation
map00250 | Alanine, aspartate and glutamate metabolism
map00280 | Valine, leucine and isoleucine degradation
map04712 | Circadian rhythm − plant
Pathway
0.2
0.3
0.4
0.5
0.6
Rich Factor
Gene Number
30
60
90
120
P.value
7.69e−04
2.58e−02
5.09e−02

d.

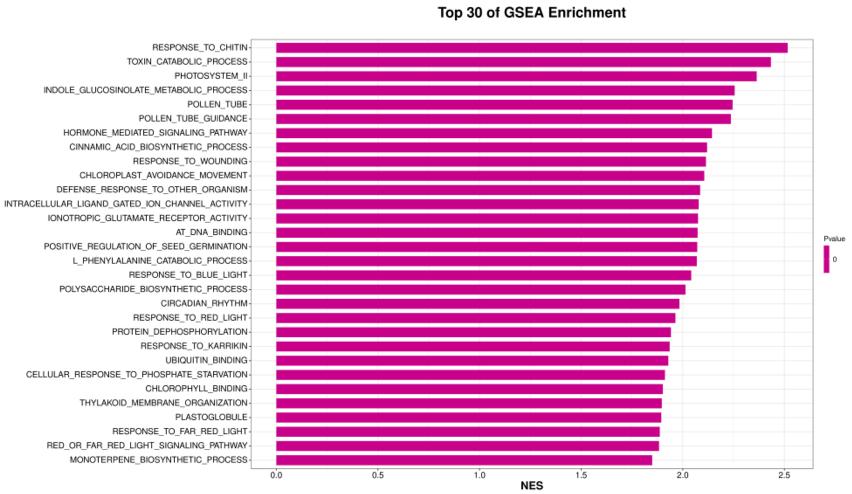

e.

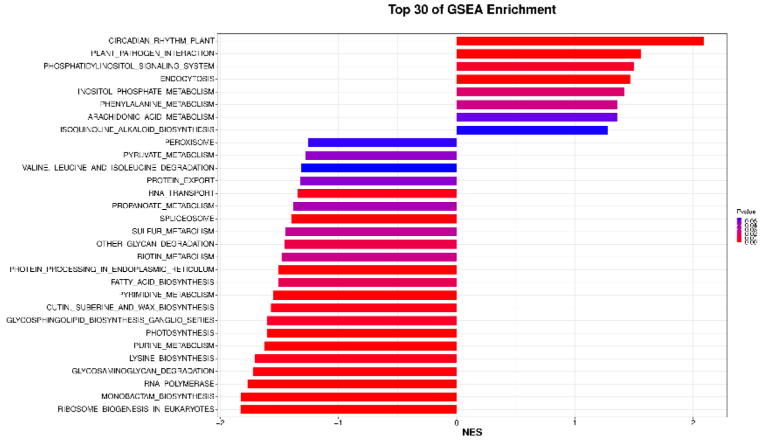

f.

Supplemental Figure S8. The volcano plot of gene expression pattern (a), the GO enrichment bar plot (b), the GO enrichment scatter plot (c), the KEGG enrichment scatter plot (d), the GO Gsea enrichment analysis (e) and the KEGG Gsea enrichment analysis (f) of DEGs between leaf tissue B and C group.

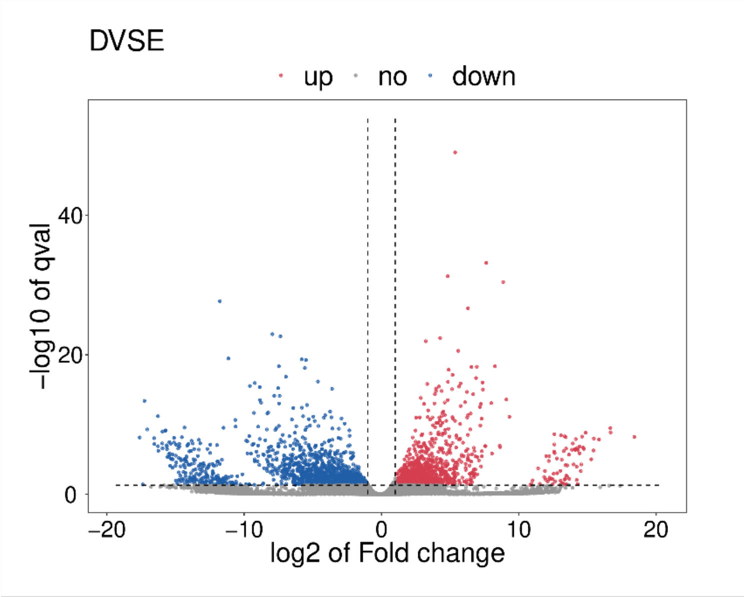
DVSE
up
no
down
-log10 of qval
40
20
0
-20
-10
0
10
20
log2 of Fold change

a.

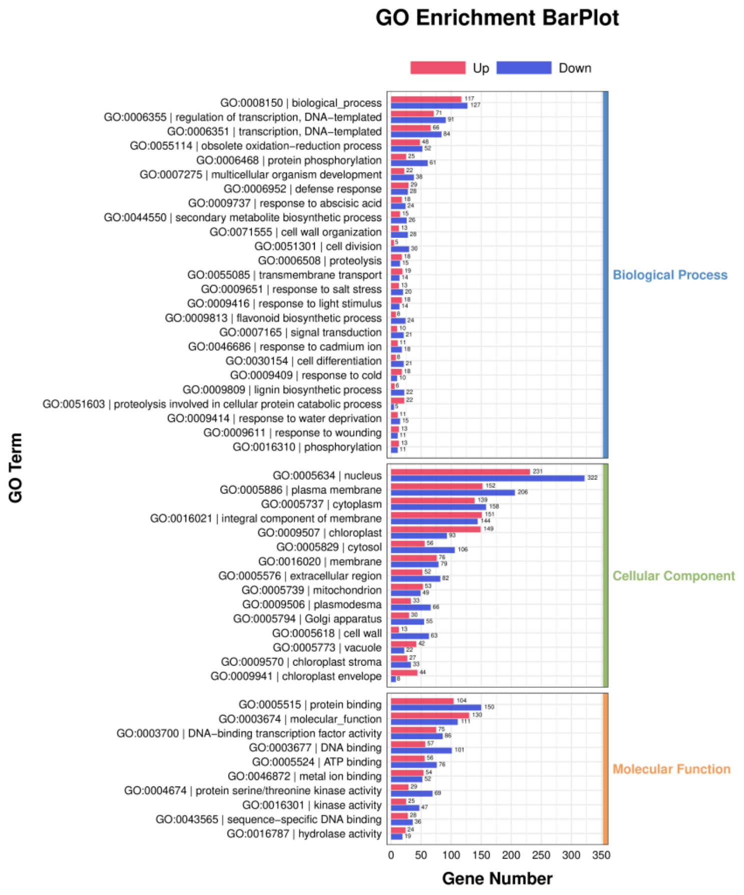
GO Enrichment BarPlot
Up
Down
GO Term
GO:0008150 | biological_process
GO:0006355 | regulation of transcription, DNA-templated
GO:0006351 | transcription, DNA-templated
GO:0055114 | obsolete oxidation-reduction process
GO:0006468 | protein phosphorylation
GO:0007275 | multicellular organism development
GO:0006952 | defense response
GO:0009737 | response to abscisic acid
GO:0044550 | secondary metabolite biosynthetic process
GO:0071555 | cell wall organization
GO:0051301 | cell division
GO:0006508 | proteolysis
GO:0055085 | transmembrane transport
GO:0009651 | response to salt stress
GO:0009416 | response to light stimulus
GO:0009813 | flavonoid biosynthetic process
GO:0007165 | signal transduction
GO:0046686 | response to cadmium ion
GO:0030154 | cell differentiation
GO:0009409 | response to cold
GO:0009809 | lignin biosynthetic process
GO:0051603 | proteolysis involved in cellular protein catabolic process
GO:0009414 | response to water deprivation
GO:0009611 | response to wounding
GO:0016310 | phosphorylation
GO:0005634 | nucleus
GO:0005886 | plasma membrane
GO:0005737 | cytoplasm
GO:0016021 | integral component of membrane
GO:0009507 | chloroplast
GO:0005829 | cytosol
GO:0016020 | membrane
GO:0005576 | extracellular region
GO:0005739 | mitochondrion
GO:0009506 | plasmodesma
GO:0005794 | Golgi apparatus
GO:0005618 | cell wall
GO:0005773 | vacuole
GO:0009570 | chloroplast stroma
GO:0009941 | chloroplast envelope
GO:0005515 | protein binding
GO:0003674 | molecular_function
GO:0003700 | DNA-binding transcription factor activity
GO:0003677 | DNA binding
GO:0005524 | ATP binding
GO:0046872 | metal ion binding
GO:0004674 | protein serine/threonine kinase activity
GO:0016301 | kinase activity
GO:0043565 | sequence-specific DNA binding
GO:0016787 | hydrolase activity
Biological Process
Cellular Component
Molecular Function
0
50
100
150
200
250
300
350
Gene Number

b.

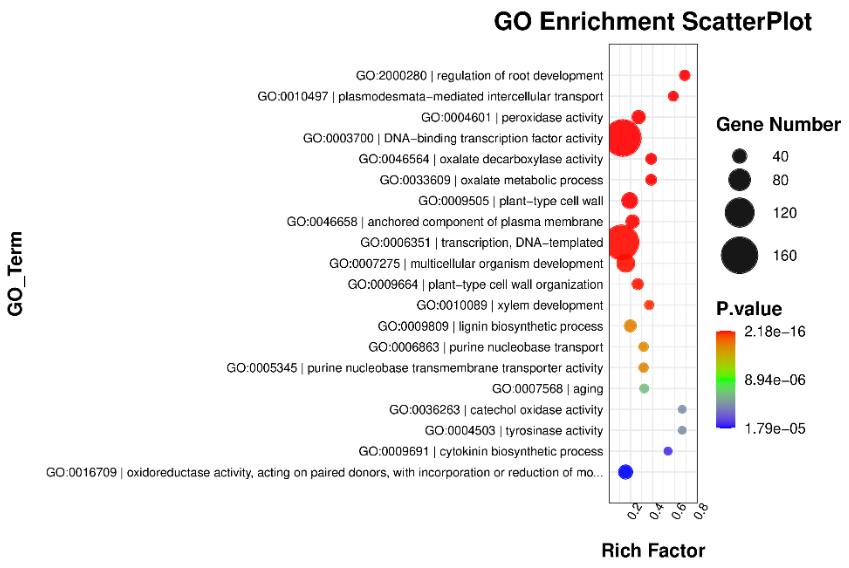
GO Enrichment ScatterPlot
GO:2000280 | regulation of root development
GO:0010497 | plasmodesmata−mediated intercellular transport
GO:0004601 | peroxidase activity
GO:0003700 | DNA−binding transcription factor activity
GO:0046564 | oxalate decarboxylase activity
GO:0033609 | oxalate metabolic process
GO:0009505 | plant−type cell wall
GO:0046658 | anchored component of plasma membrane
GO:0006351 | transcription, DNA−templated
GO:0007275 | multicellular organism development
GO:0009664 | plant−type cell wall organization
GO:0010089 | xylem development
GO:0009809 | lignin biosynthetic process
GO:0006863 | purine nucleobase transport
GO:0005345 | purine nucleobase transmembrane transporter activity
GO:0007568 | aging
GO:0036263 | catechol oxidase activity
GO:0004503 | tyrosinase activity
GO:0009691 | cytokinin biosynthetic process
GO:0016709 | oxidoreductase activity, acting on paired donors, with incorporation or reduction of mo...
GO_Term
0.2
0.4
0.6
0.8
Rich Factor
Gene Number
40
80
120
160
P.value
2.18e−16
8.94e−06
1.79e−05

c.

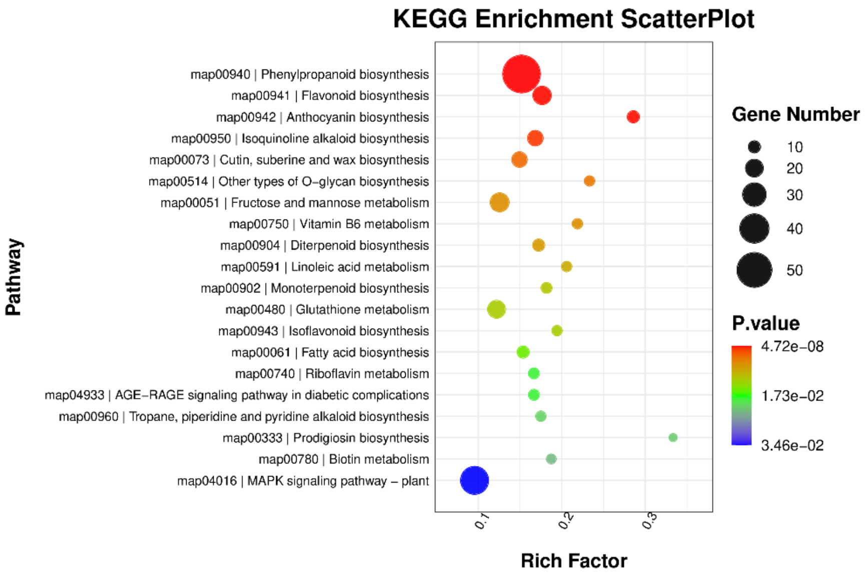
KEGG Enrichment ScatterPlot
map00940 | Phenylpropanoid biosynthesis
map00941 | Flavonoid biosynthesis
map00942 | Anthocyanin biosynthesis
map00950 | Isoquinoline alkaloid biosynthesis
map00073 | Cutin, suberine and wax biosynthesis
map00514 | Other types of O−glycan biosynthesis
map00051 | Fructose and mannose metabolism
map00750 | Vitamin B6 metabolism
map00904 | Diterpenoid biosynthesis
map00591 | Linoleic acid metabolism
map00902 | Monoterpenoid biosynthesis
map00480 | Glutathione metabolism
map00943 | Isoflavonoid biosynthesis
map00061 | Fatty acid biosynthesis
map00740 | Riboflavin metabolism
map04933 | AGE−RAGE signaling pathway in diabetic complications
map00960 | Tropane, piperidine and pyridine alkaloid biosynthesis
map00333 | Prodigiosin biosynthesis
map00780 | Biotin metabolism
map04016 | MAPK signaling pathway – plant
Pathway
0.1
0.2
0.3
Rich Factor
Gene Number
10
20
30
40
50
P.value
4.72e−08
1.73e−02
3.46e−02

d.

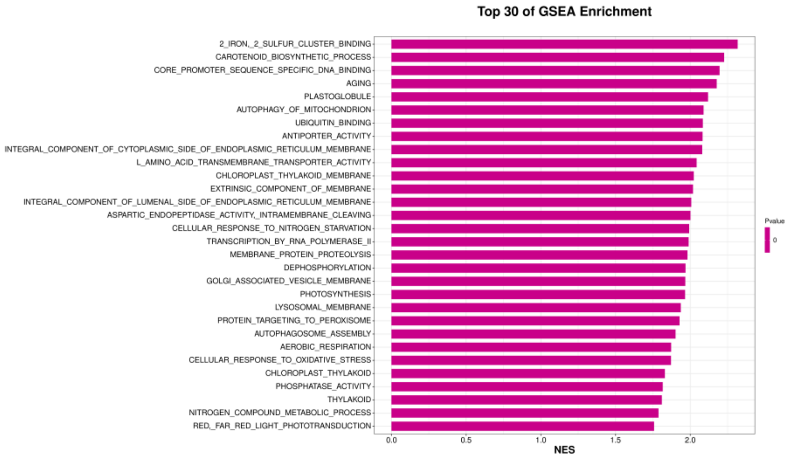

e.

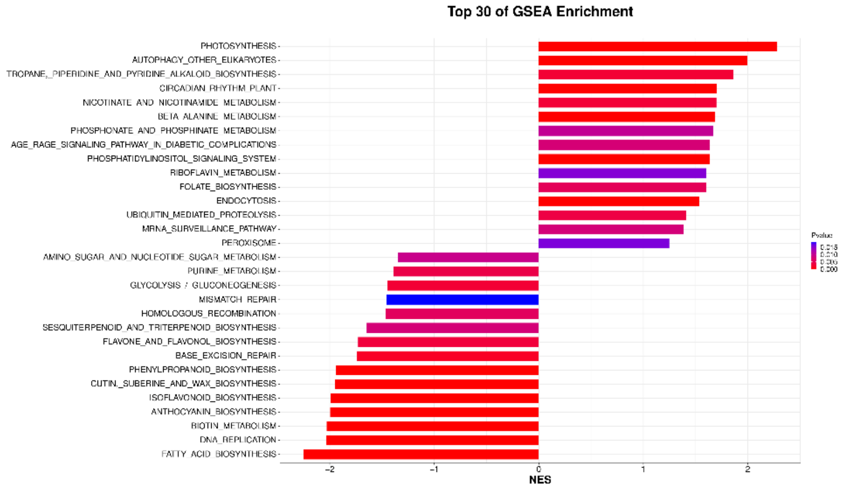

f.

Supplemental Figure S9. The volcano plot of gene expression pattern (a), the GO enrichment bar plot (b), the GO enrichment scatter plot (c), the KEGG enrichment scatter plot (d), the GO Gsea enrichment analysis (e) and the KEGG Gsea enrichment analysis (f) of DEGs between flower bud tissue D and E group.

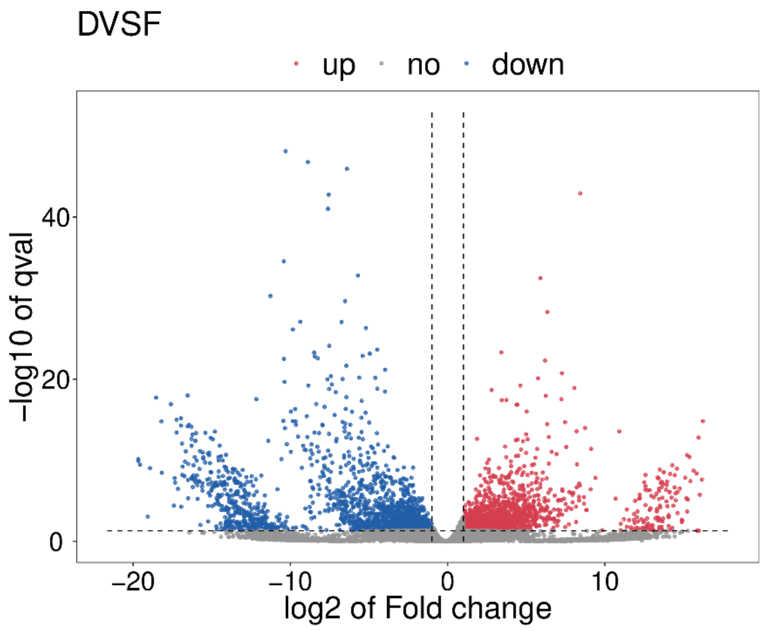
DVSF
up
no
down
−log10 of qval
log2 of Fold change
0
20
40
−20
−10
0
10

a.

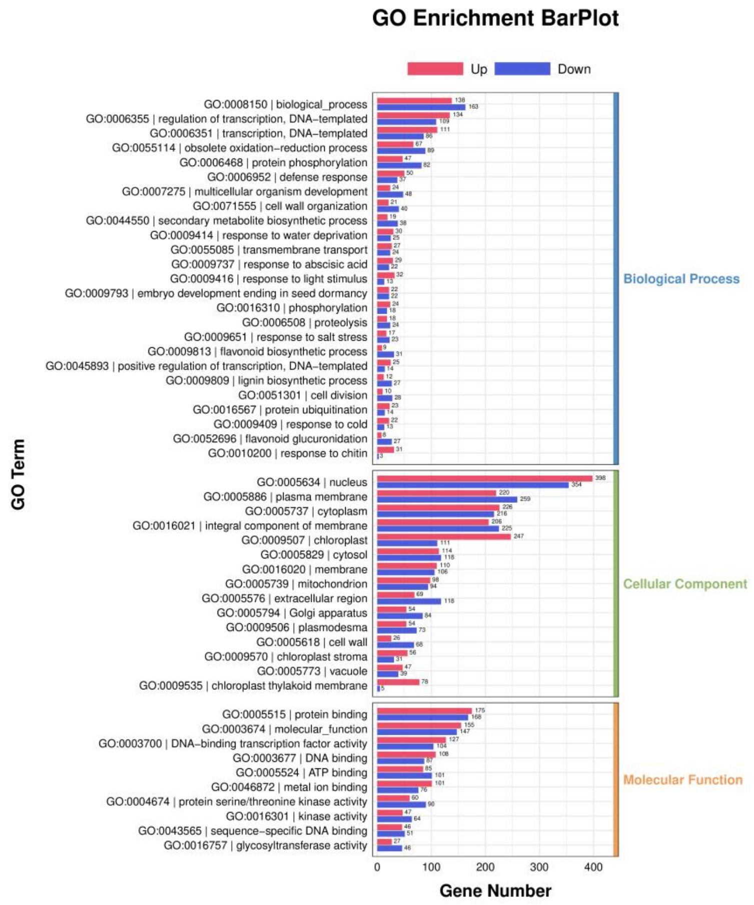
GO Enrichment BarPlot
Up
Down
GO Term
GO:0008150 | biological_process
GO:0006355 | regulation of transcription, DNA−templated
GO:0006351 | transcription, DNA−templated
GO:0055114 | obsolete oxidation−reduction process
GO:0006468 | protein phosphorylation
GO:0006952 | defense response
GO:0007275 | multicellular organism development
GO:0071555 | cell wall organization
GO:0044550 | secondary metabolite biosynthetic process
GO:0009414 | response to water deprivation
GO:0055085 | transmembrane transport
GO:0009737 | response to abscisic acid
GO:0009416 | response to light stimulus
GO:0009793 | embryo development ending in seed dormancy
GO:0016310 | phosphorylation
GO:0006508 | proteolysis
GO:0009651 | response to salt stress
GO:0009813 | flavonoid biosynthetic process
GO:0045893 | positive regulation of transcription, DNA−templated
GO:0009809 | lignin biosynthetic process
GO:0051301 | cell division
GO:0016567 | protein ubiquitination
GO:0009409 | response to cold
GO:0052696 | flavonoid glucuronidation
GO:0010200 | response to chitin
GO:0005634 | nucleus
GO:0005886 | plasma membrane
GO:0005737 | cytoplasm
GO:0016021 | integral component of membrane
GO:0009507 | chloroplast
GO:0005829 | cytosol
GO:0016020 | membrane
GO:0005739 | mitochondrion
GO:0005576 | extracellular region
GO:0005794 | Golgi apparatus
GO:0009506 | plasmodesma
GO:0005618 | cell wall
GO:0009570 | chloroplast stroma
GO:0005773 | vacuole
GO:0009535 | chloroplast thylakoid membrane
GO:0005515 | protein binding
GO:0003674 | molecular_function
GO:0003700 | DNA−binding transcription factor activity
GO:0003677 | DNA binding
GO:0005524 | ATP binding
GO:0046872 | metal ion binding
GO:0004674 | protein serine/threonine kinase activity
GO:0016301 | kinase activity
GO:0043565 | sequence−specific DNA binding
GO:0016757 | glycosyltransferase activity
Biological Process
Cellular Component
Molecular Function
0
100
200
300
400
Gene Number

b.

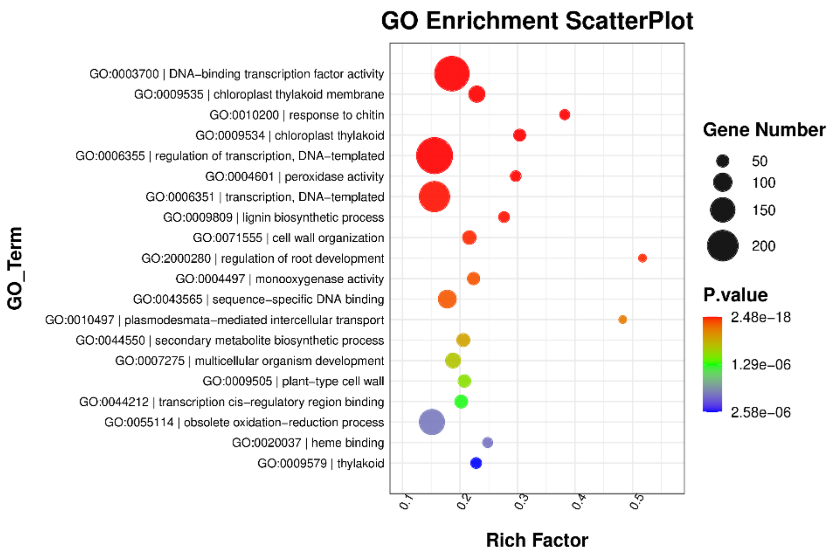
GO Enrichment ScatterPlot
GO:0003700 | DNA−binding transcription factor activity
GO:0009535 | chloroplast thylakoid membrane
GO:0010200 | response to chitin
GO:0009534 | chloroplast thylakoid
GO:0006355 | regulation of transcription, DNA−templated
GO:0004601 | peroxidase activity
GO:0006351 | transcription, DNA−templated
GO:0009809 | lignin biosynthetic process
GO:0071555 | cell wall organization
GO:2000280 | regulation of root development
GO:0004497 | monooxygenase activity
GO:0043565 | sequence−specific DNA binding
GO:0010497 | plasmodesmata−mediated intercellular transport
GO:0044550 | secondary metabolite biosynthetic process
GO:0007275 | multicellular organism development
GO:0009505 | plant−type cell wall
GO:0044212 | transcription cis−regulatory region binding
GO:0055114 | obsolete oxidation−reduction process
GO:0020037 | heme binding
GO:0009579 | thylakoid
GO_Term
0.1
0.2
0.3
0.4
0.5
Rich Factor
Gene Number
50
100
150
200
P.value
2.48e−18
1.29e−06
2.58e−06

c.

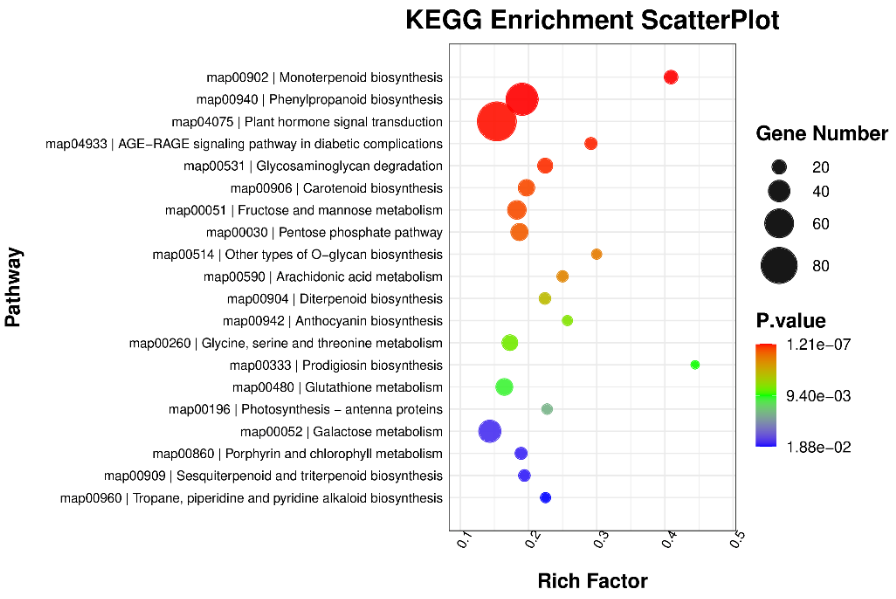
KEGG Enrichment ScatterPlot
map00902 | Monoterpenoid biosynthesis
map00940 | Phenylpropanoid biosynthesis
map04075 | Plant hormone signal transduction
map04933 | AGE−RAGE signaling pathway in diabetic complications
map00531 | Glycosaminoglycan degradation
map00906 | Carotenoid biosynthesis
map00051 | Fructose and mannose metabolism
map00030 | Pentose phosphate pathway
map00514 | Other types of O−glycan biosynthesis
map00590 | Arachidonic acid metabolism
map00904 | Diterpenoid biosynthesis
map00942 | Anthocyanin biosynthesis
map00260 | Glycine, serine and threonine metabolism
map00333 | Prodigiosin biosynthesis
map00480 | Glutathione metabolism
map00196 | Photosynthesis − antenna proteins
map00052 | Galactose metabolism
map00860 | Porphyrin and chlorophyll metabolism
map00909 | Sesquiterpenoid and triterpenoid biosynthesis
map00960 | Tropane, piperidine and pyridine alkaloid biosynthesis
Pathway
0.1
0.2
0.3
0.4
0.5
Rich Factor
Gene Number
20
40
60
80
P.value
1.21e−07
9.40e−03
1.88e−02

d.

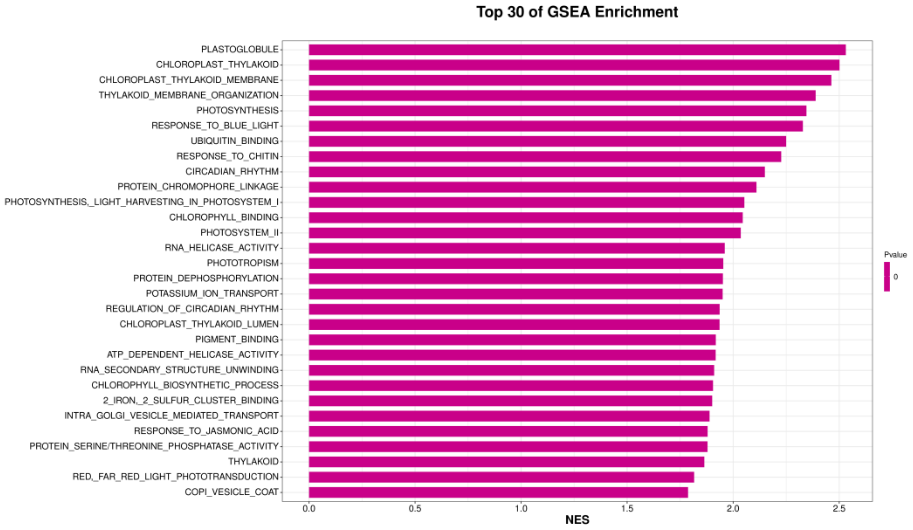

e.

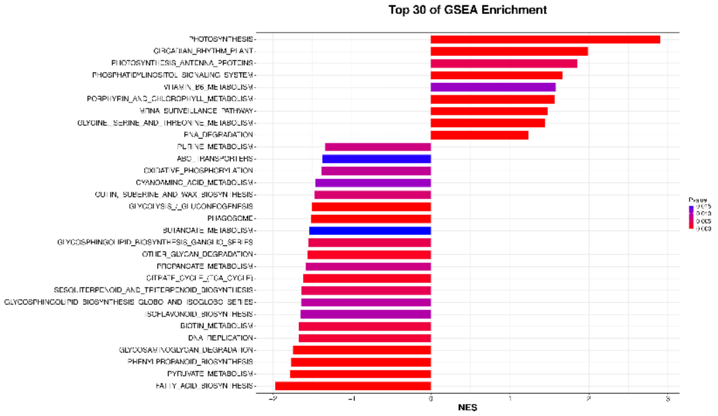

f.

Supplemental Figure S10. The volcano plot of gene expression pattern (a), the GO enrichment bar plot (b), the GO enrichment scatter plot (c), the KEGG enrichment scatter plot (d), the GO Gsea enrichment analysis (e) and the KEGG Gsea enrichment analysis (f) of DEGs between flower bud tissue D and F group.

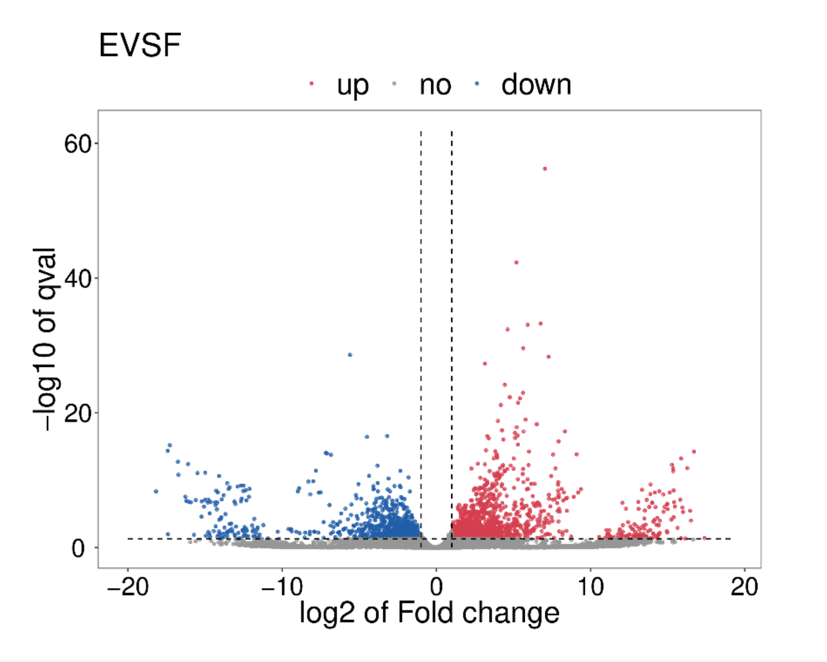

EVSF
up
no
down
−log10 of qval
60
40
20
0
−20
−10
0
10
20
log2 of Fold change

a.

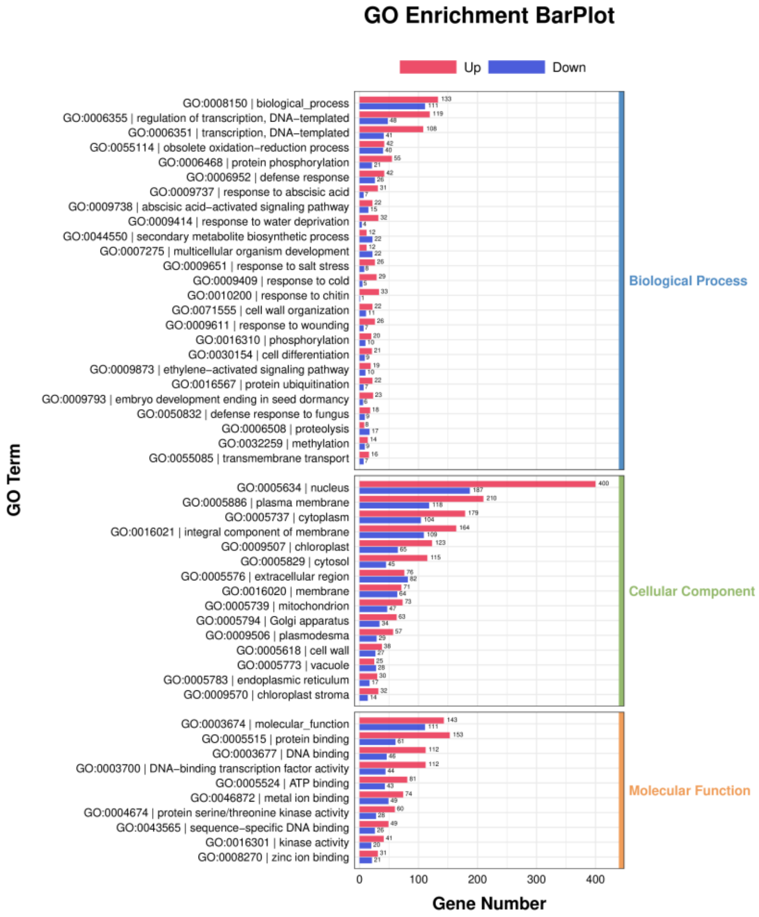

GO Enrichment BarPlot
Up
Down
GO Term
GO:0008150 | biological_process
GO:0006355 | regulation of transcription, DNA−templated
GO:0006351 | transcription, DNA−templated
GO:0055114 | obsolete oxidation−reduction process
GO:0006468 | protein phosphorylation
GO:0006952 | defense response
GO:0009737 | response to abscisic acid
GO:0009738 | abscisic acid−activated signaling pathway
GO:0009414 | response to water deprivation
GO:0044550 | secondary metabolite biosynthetic process
GO:0007275 | multicellular organism development
GO:0009651 | response to salt stress
GO:0009409 | response to cold
GO:0010200 | response to chitin
GO:0071555 | cell wall organization
GO:0009611 | response to wounding
GO:0016310 | phosphorylation
GO:0030154 | cell differentiation
GO:0009873 | ethylene−activated signaling pathway
GO:0016567 | protein ubiquitination
GO:0009793 | embryo development ending in seed dormancy
GO:0050832 | defense response to fungus
GO:0006508 | proteolysis
GO:0032259 | methylation
GO:0055085 | transmembrane transport
GO:0005634 | nucleus
GO:0005886 | plasma membrane
GO:0005737 | cytoplasm
GO:0016021 | integral component of membrane
GO:0009507 | chloroplast
GO:0005829 | cytosol
GO:0005576 | extracellular region
GO:0016020 | membrane
GO:0005739 | mitochondrion
GO:0005794 | Golgi apparatus
GO:0009506 | plasmodesma
GO:0005618 | cell wall
GO:0005773 | vacuole
GO:0005783 | endoplasmic reticulum
GO:0009570 | chloroplast stroma
GO:0003674 | molecular_function
GO:0005515 | protein binding
GO:0003677 | DNA binding
GO:0003700 | DNA−binding transcription factor activity
GO:0005524 | ATP binding
GO:0046872 | metal ion binding
GO:0004674 | protein serine/threonine kinase activity
GO:0043565 | sequence−specific DNA binding
GO:0016301 | kinase activity
GO:0008270 | zinc ion binding
Biological Process
Cellular Component
Molecular Function
0
100
200
300
400
Gene Number

b.

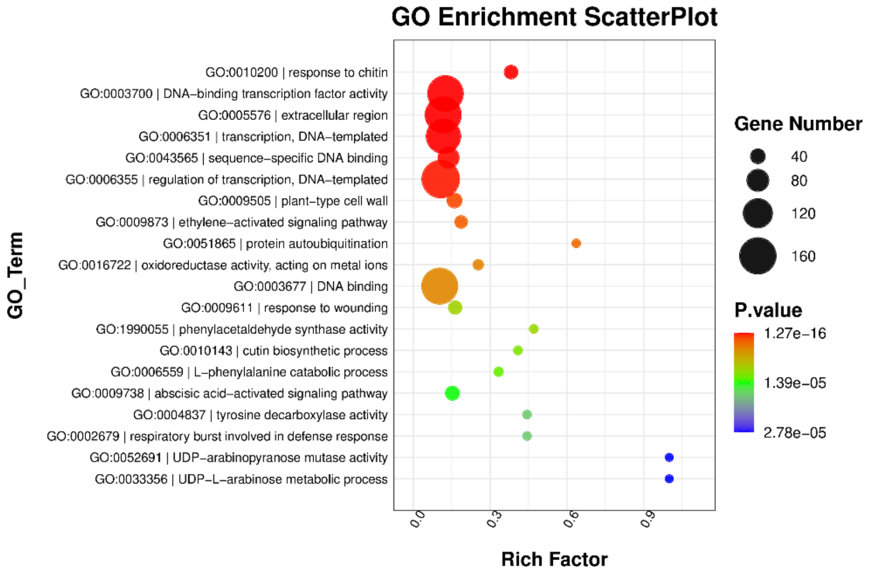
GO Enrichment ScatterPlot
GO:0010200 | response to chitin
GO:0003700 | DNA–binding transcription factor activity
GO:0005576 | extracellular region
GO:0006351 | transcription, DNA–templated
GO:0043565 | sequence–specific DNA binding
GO:0006355 | regulation of transcription, DNA–templated
GO:0009505 | plant–type cell wall
GO:0009873 | ethylene–activated signaling pathway
GO:0051865 | protein autoubiquitination
GO:0016722 | oxidoreductase activity, acting on metal ions
GO:0003677 | DNA binding
GO:0009611 | response to wounding
GO:1990055 | phenylacetaldehyde synthase activity
GO:0010143 | cutin biosynthetic process
GO:0006559 | L–phenylalanine catabolic process
GO:0009738 | abscisic acid–activated signaling pathway
GO:0004837 | tyrosine decarboxylase activity
GO:0002679 | respiratory burst involved in defense response
GO:0052691 | UDP–arabinopyranose mutase activity
GO:0033356 | UDP–L–arabinose metabolic process
GO_Term
0.0
0.3
0.6
0.9
Rich Factor
Gene Number
40
80
120
160
P.value
1.27e–16
1.39e–05
2.78e–05

b.

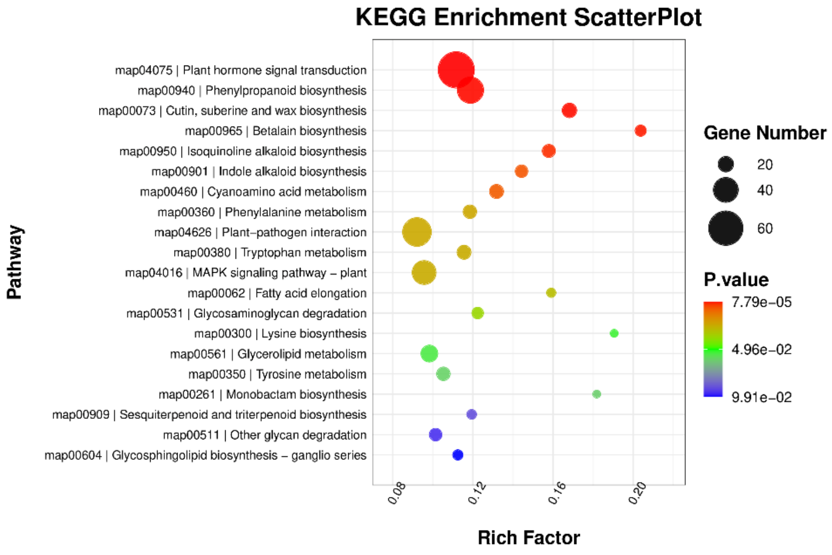
KEGG Enrichment ScatterPlot
map04075 | Plant hormone signal transduction
map00940 | Phenylpropanoid biosynthesis
map00073 | Cutin, suberine and wax biosynthesis
map00965 | Betalain biosynthesis
map00950 | Isoquinoline alkaloid biosynthesis
map00901 | Indole alkaloid biosynthesis
map00460 | Cyanoamino acid metabolism
map00360 | Phenylalanine metabolism
map04626 | Plant–pathogen interaction
map00380 | Tryptophan metabolism
map04016 | MAPK signaling pathway – plant
map00062 | Fatty acid elongation
map00531 | Glycosaminoglycan degradation
map00300 | Lysine biosynthesis
map00561 | Glycerolipid metabolism
map00350 | Tyrosine metabolism
map00261 | Monobactam biosynthesis
map00909 | Sesquiterpenoid and triterpenoid biosynthesis
map00511 | Other glycan degradation
map00604 | Glycosphingolipid biosynthesis – ganglio series
Pathway
0.08
0.12
0.16
0.20
Rich Factor
Gene Number
20
40
60
P.value
7.79e–05
4.96e–02
9.91e–02

d.

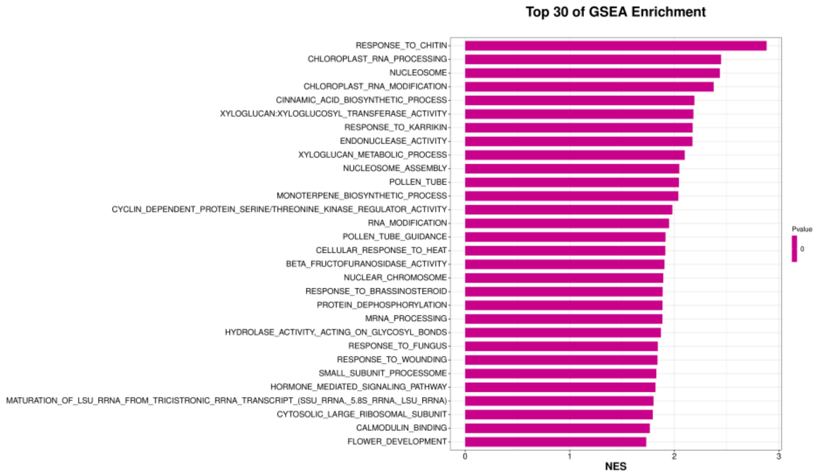

e.

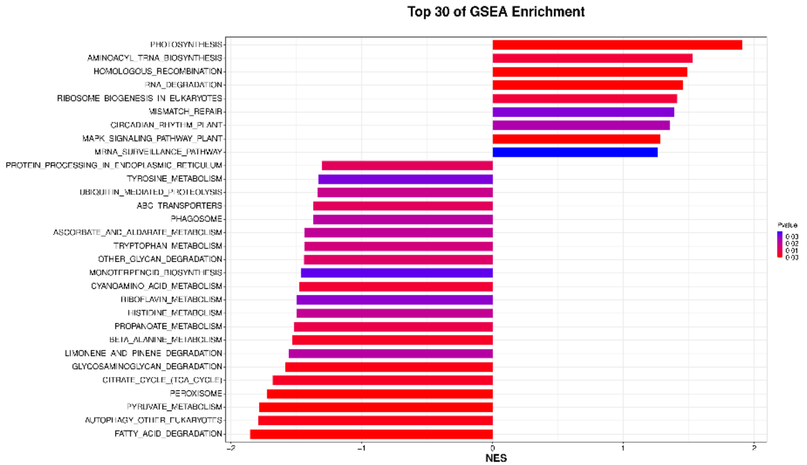

f.

Supplemental Figure S11. The volcano plot of gene expression pattern (a), the GO enrichment bar plot (b), the GO enrichment scatter plot (c), the KEGG enrichment scatter plot (d), the GO Gsea enrichment analysis (e) and the KEGG Gsea enrichment analysis (f) of DEGs between flower bud tissue E and F group.

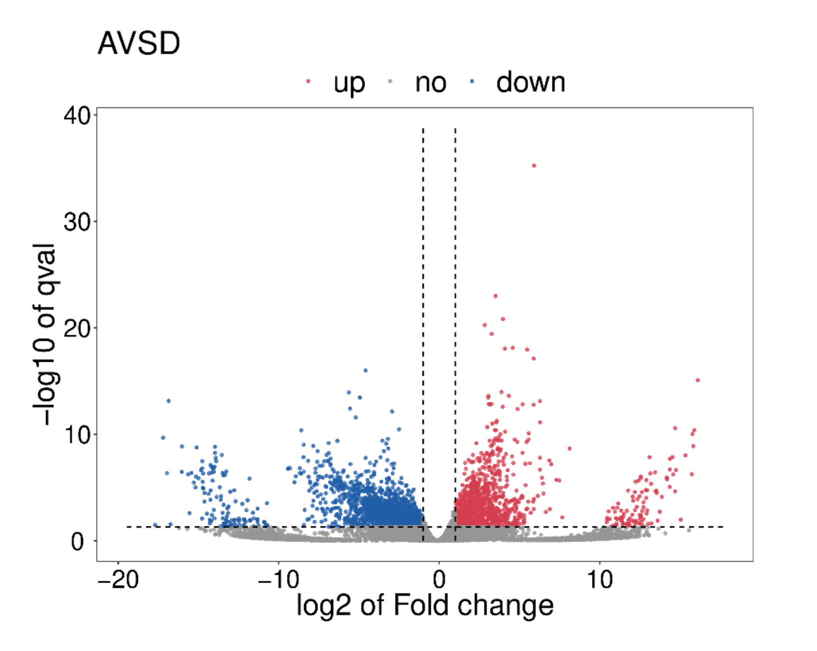
AVSD
up
no
down
−log10 of qval
log2 of Fold change
40
30
20
10
0
−20
−10
0
10

a.

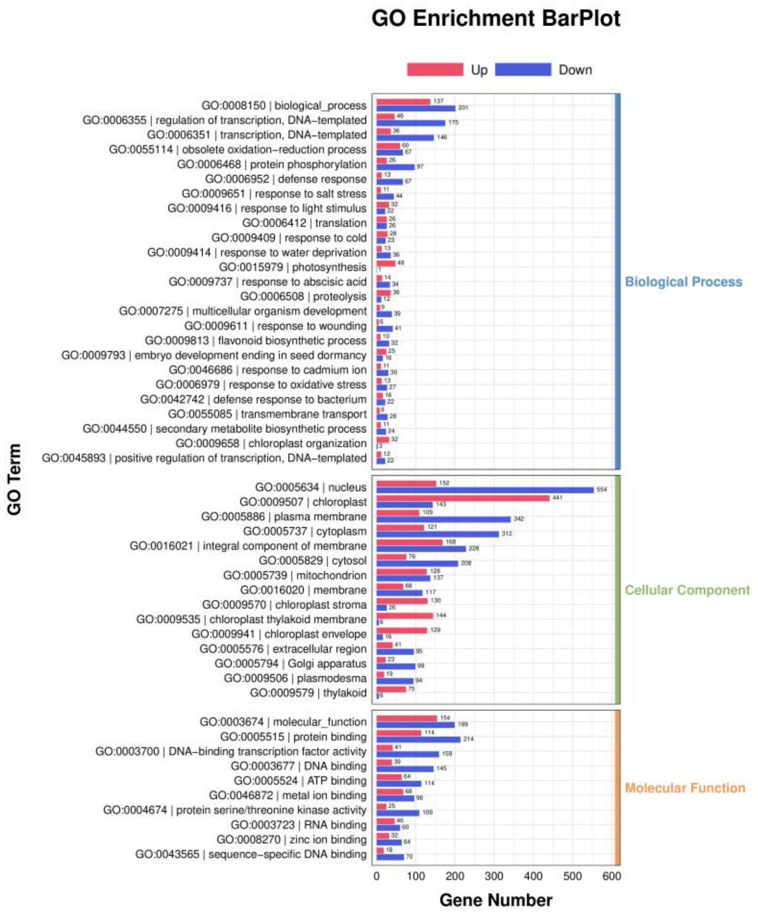
GO Enrichment BarPlot
Up
Down
GO Term
GO:0008150 | biological_process
GO:0006355 | regulation of transcription, DNA−templated
GO:0006351 | transcription, DNA−templated
GO:0055114 | obsolete oxidation−reduction process
GO:0006468 | protein phosphorylation
GO:0006952 | defense response
GO:0009651 | response to salt stress
GO:0009416 | response to light stimulus
GO:0006412 | translation
GO:0009409 | response to cold
GO:0009414 | response to water deprivation
GO:0015979 | photosynthesis
GO:0009737 | response to abscisic acid
GO:0006508 | proteolysis
GO:0007275 | multicellular organism development
GO:0009611 | response to wounding
GO:0009813 | flavonoid biosynthetic process
GO:0009793 | embryo development ending in seed dormancy
GO:0046686 | response to cadmium ion
GO:0006979 | response to oxidative stress
GO:0042742 | defense response to bacterium
GO:0055085 | transmembrane transport
GO:0044550 | secondary metabolite biosynthetic process
GO:0009658 | chloroplast organization
GO:0045893 | positive regulation of transcription, DNA−templated
GO:0005634 | nucleus
GO:0009507 | chloroplast
GO:0005886 | plasma membrane
GO:0005737 | cytoplasm
GO:0016021 | integral component of membrane
GO:0005829 | cytosol
GO:0005739 | mitochondrion
GO:0016020 | membrane
GO:0009570 | chloroplast stroma
GO:0009535 | chloroplast thylakoid membrane
GO:0009941 | chloroplast envelope
GO:0005576 | extracellular region
GO:0005794 | Golgi apparatus
GO:0009506 | plasmodesma
GO:0009579 | thylakoid
GO:0003674 | molecular_function
GO:0005515 | protein binding
GO:0003700 | DNA−binding transcription factor activity
GO:0003677 | DNA binding
GO:0005524 | ATP binding
GO:0046872 | metal ion binding
GO:0004674 | protein serine/threonine kinase activity
GO:0003723 | RNA binding
GO:0008270 | zinc ion binding
GO:0043565 | sequence−specific DNA binding
Biological Process
Cellular Component
Molecular Function
0
100
200
300
400
500
600
Gene Number

b.

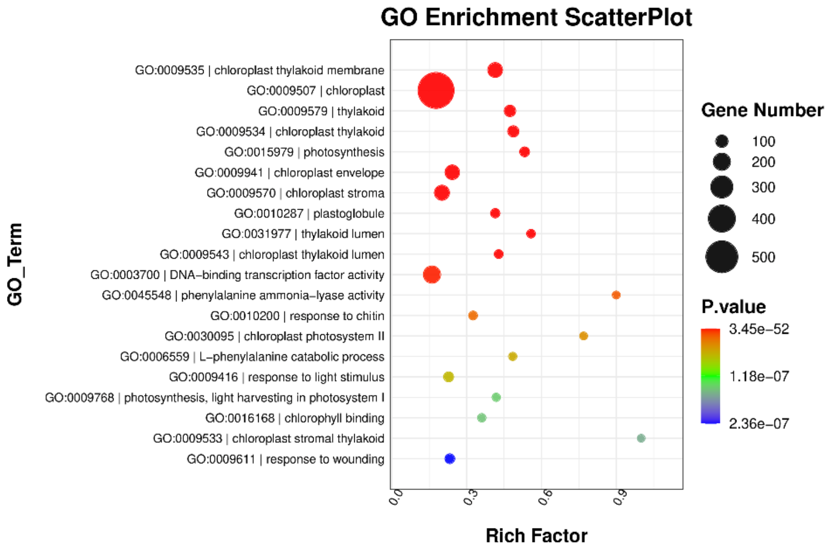
GO Enrichment ScatterPlot
GO:0009535 | chloroplast thylakoid membrane
GO:0009507 | chloroplast
GO:0009579 | thylakoid
GO:0009534 | chloroplast thylakoid
GO:0015979 | photosynthesis
GO:0009941 | chloroplast envelope
GO:0009570 | chloroplast stroma
GO:0010287 | plastoglobule
GO:0031977 | thylakoid lumen
GO:0009543 | chloroplast thylakoid lumen
GO:0003700 | DNA–binding transcription factor activity
GO:0045548 | phenylalanine ammonia–lyase activity
GO:0010200 | response to chitin
GO:0030095 | chloroplast photosystem II
GO:0006559 | L–phenylalanine catabolic process
GO:0009416 | response to light stimulus
GO:0009768 | photosynthesis, light harvesting in photosystem I
GO:0016168 | chlorophyll binding
GO:0009533 | chloroplast stromal thylakoid
GO:0009611 | response to wounding
GO_Term
Rich Factor
0.0
0.3
0.6
0.9
Gene Number
100
200
300
400
500
P.value
3.45e–52
1.18e–07
2.36e–07

c.

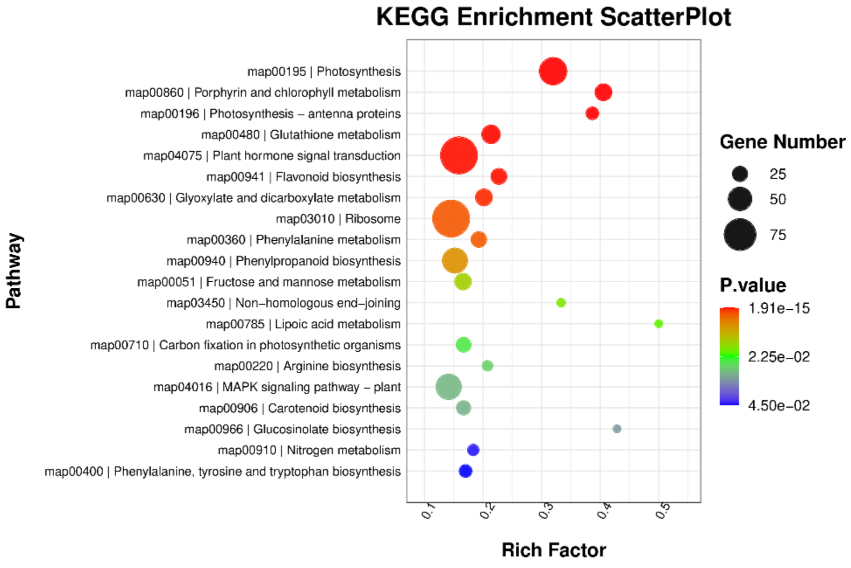
KEGG Enrichment ScatterPlot
map00195 | Photosynthesis
map00860 | Porphyrin and chlorophyll metabolism
map00196 | Photosynthesis – antenna proteins
map00480 | Glutathione metabolism
map04075 | Plant hormone signal transduction
map00941 | Flavonoid biosynthesis
map00630 | Glyoxylate and dicarboxylate metabolism
map03010 | Ribosome
map00360 | Phenylalanine metabolism
map00940 | Phenylpropanoid biosynthesis
map00051 | Fructose and mannose metabolism
map03450 | Non–homologous end–joining
map00785 | Lipoic acid metabolism
map00710 | Carbon fixation in photosynthetic organisms
map00220 | Arginine biosynthesis
map04016 | MAPK signaling pathway – plant
map00906 | Carotenoid biosynthesis
map00966 | Glucosinolate biosynthesis
map00910 | Nitrogen metabolism
map00400 | Phenylalanine, tyrosine and tryptophan biosynthesis
Pathway
Rich Factor
0.1
0.2
0.3
0.4
0.5
Gene Number
25
50
75
P.value
1.91e–15
2.25e–02
4.50e–02

d.

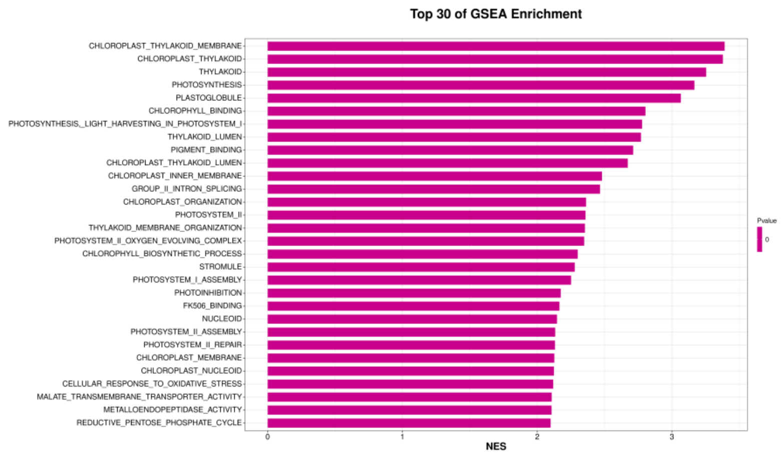

e.

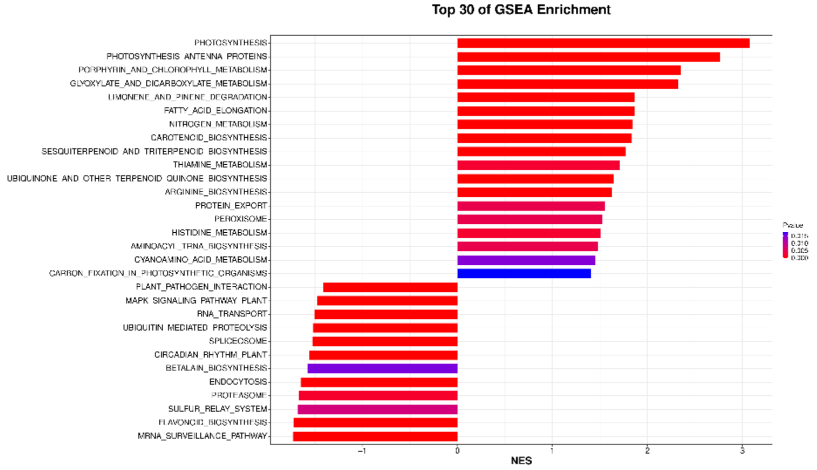

f.

Supplemental Figure S12. The volcano plot of gene expression pattern (a), the GO enrichment bar plot (b), the GO enrichment scatter plot (c), the KEGG enrichment scatter plot (d), the GO Gsea enrichment analysis (e) and the KEGG Gsea enrichment analysis (f) of DEGs between leaf tissue A and flower bud tissue D group.

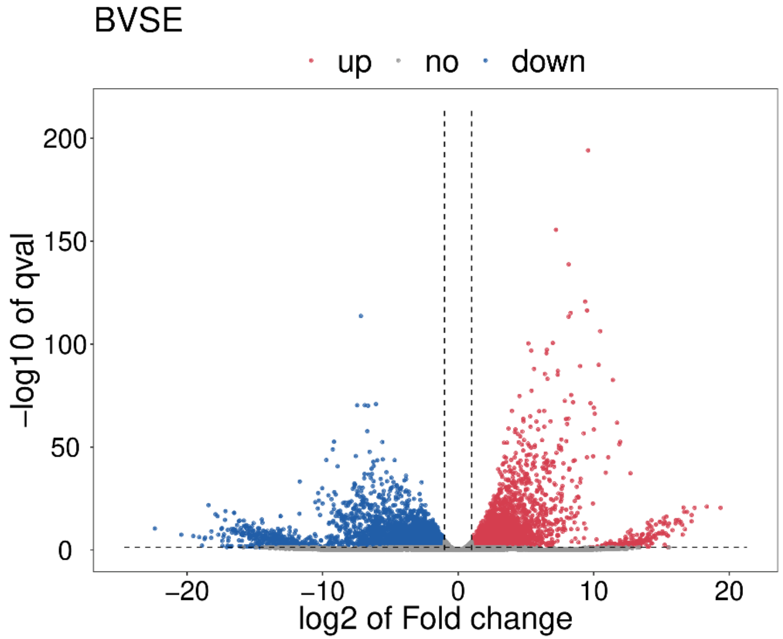
BVSE
up
no
down
200
150
100
50
0
−log10 of qval
−20
−10
0
10
20
log2 of Fold change

a.

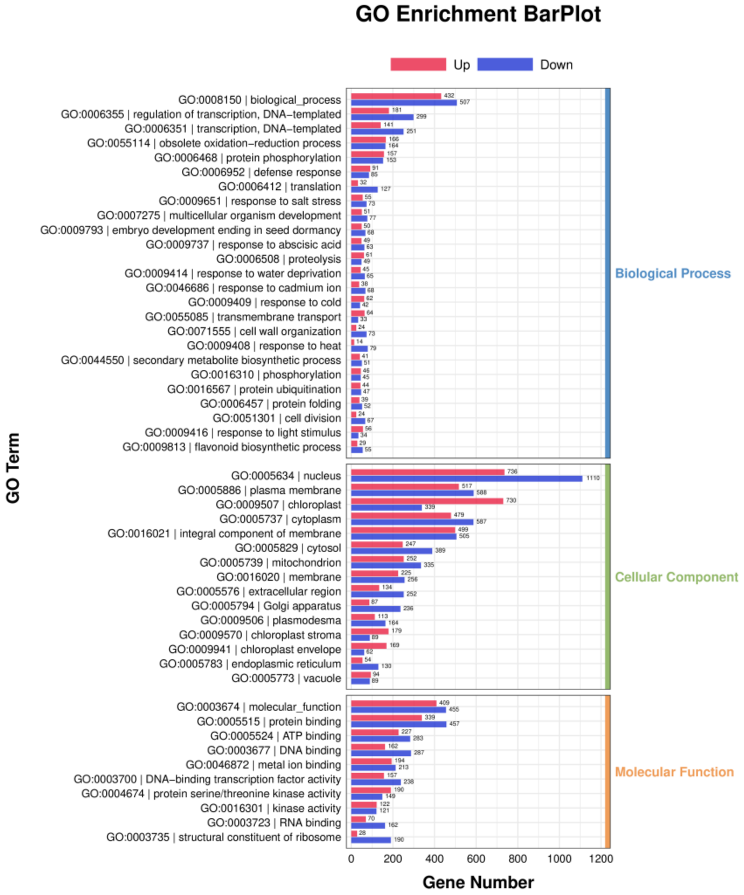
GO Enrichment BarPlot
Up
Down
GO Term
GO:0008150 | biological_process
GO:0006355 | regulation of transcription, DNA−templated
GO:0006351 | transcription, DNA−templated
GO:0055114 | obsolete oxidation−reduction process
GO:0006468 | protein phosphorylation
GO:0006952 | defense response
GO:0006412 | translation
GO:0009651 | response to salt stress
GO:0007275 | multicellular organism development
GO:0009793 | embryo development ending in seed dormancy
GO:0009737 | response to abscisic acid
GO:0006508 | proteolysis
GO:0009414 | response to water deprivation
GO:0046686 | response to cadmium ion
GO:0009409 | response to cold
GO:0055085 | transmembrane transport
GO:0071555 | cell wall organization
GO:0009408 | response to heat
GO:0044550 | secondary metabolite biosynthetic process
GO:0016310 | phosphorylation
GO:0016567 | protein ubiquitination
GO:0006457 | protein folding
GO:0051301 | cell division
GO:0009416 | response to light stimulus
GO:0009813 | flavonoid biosynthetic process
Biological Process
GO:0005634 | nucleus
GO:0005886 | plasma membrane
GO:0009507 | chloroplast
GO:0005737 | cytoplasm
GO:0016021 | integral component of membrane
GO:0005829 | cytosol
GO:0005739 | mitochondrion
GO:0016020 | membrane
GO:0005576 | extracellular region
GO:0005794 | Golgi apparatus
GO:0009506 | plasmodesma
GO:0009570 | chloroplast stroma
GO:0009941 | chloroplast envelope
GO:0005783 | endoplasmic reticulum
GO:0005773 | vacuole
Cellular Component
GO:0003674 | molecular_function
GO:0005515 | protein binding
GO:0005524 | ATP binding
GO:0003677 | DNA binding
GO:0046872 | metal ion binding
GO:0003700 | DNA−binding transcription factor activity
GO:0004674 | protein serine/threonine kinase activity
GO:0016301 | kinase activity
GO:0003723 | RNA binding
GO:0003735 | structural constituent of ribosome
Molecular Function
0
200
400
600
800
1000
1200
Gene Number

b.

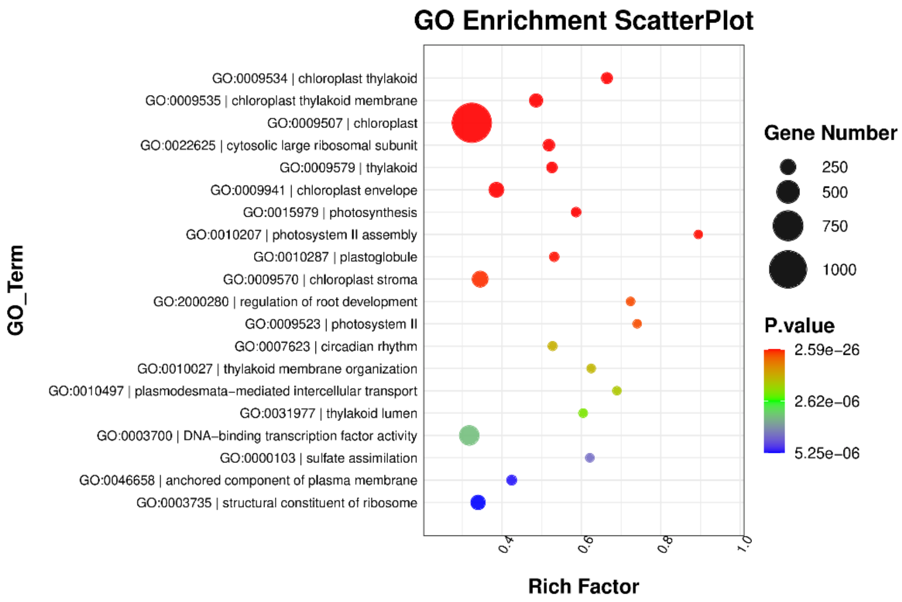
GO Enrichment ScatterPlot
GO:0009534 | chloroplast thylakoid
GO:0009535 | chloroplast thylakoid membrane
GO:0009507 | chloroplast
GO:0022625 | cytosolic large ribosomal subunit
GO:0009579 | thylakoid
GO:0009941 | chloroplast envelope
GO:0015979 | photosynthesis
GO:0010207 | photosystem II assembly
GO:0010287 | plastoglobule
GO:0009570 | chloroplast stroma
GO:2000280 | regulation of root development
GO:0009523 | photosystem II
GO:0007623 | circadian rhythm
GO:0010027 | thylakoid membrane organization
GO:0010497 | plasmodesmata−mediated intercellular transport
GO:0031977 | thylakoid lumen
GO:0003700 | DNA−binding transcription factor activity
GO:0000103 | sulfate assimilation
GO:0046658 | anchored component of plasma membrane
GO:0003735 | structural constituent of ribosome
GO_Term
0.4
0.6
0.8
1.0
Rich Factor
Gene Number
250
500
750
1000
P.value
2.59e−26
2.62e−06
5.25e−06

c.

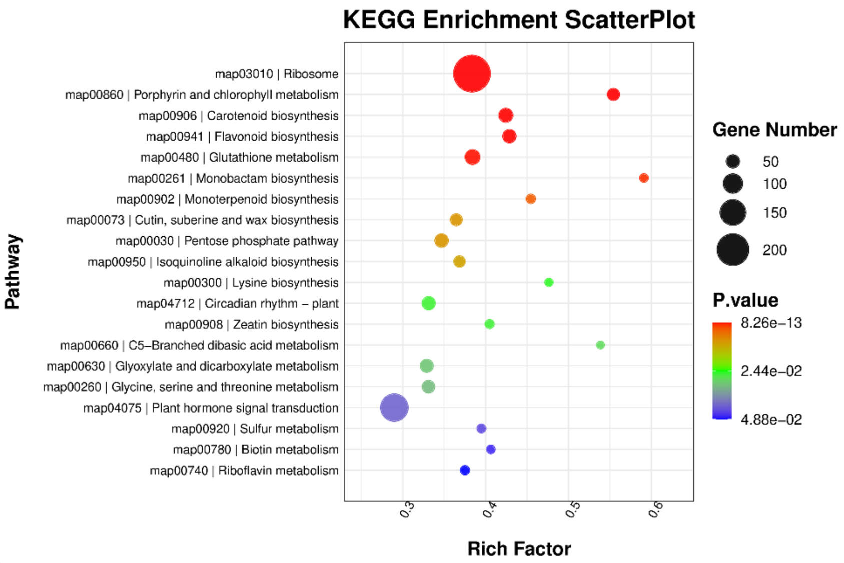
KEGG Enrichment ScatterPlot
map03010 | Ribosome
map00860 | Porphyrin and chlorophyll metabolism
map00906 | Carotenoid biosynthesis
map00941 | Flavonoid biosynthesis
map00480 | Glutathione metabolism
map00261 | Monobactam biosynthesis
map00902 | Monoterpenoid biosynthesis
map00073 | Cutin, suberine and wax biosynthesis
map00030 | Pentose phosphate pathway
map00950 | Isoquinoline alkaloid biosynthesis
map00300 | Lysine biosynthesis
map04712 | Circadian rhythm − plant
map00908 | Zeatin biosynthesis
map00660 | C5−Branched dibasic acid metabolism
map00630 | Glyoxylate and dicarboxylate metabolism
map00260 | Glycine, serine and threonine metabolism
map04075 | Plant hormone signal transduction
map00920 | Sulfur metabolism
map00780 | Biotin metabolism
map00740 | Riboflavin metabolism
Pathway
0.3
0.4
0.5
0.6
Rich Factor
Gene Number
50
100
150
200
P.value
8.26e−13
2.44e−02
4.88e−02

d.

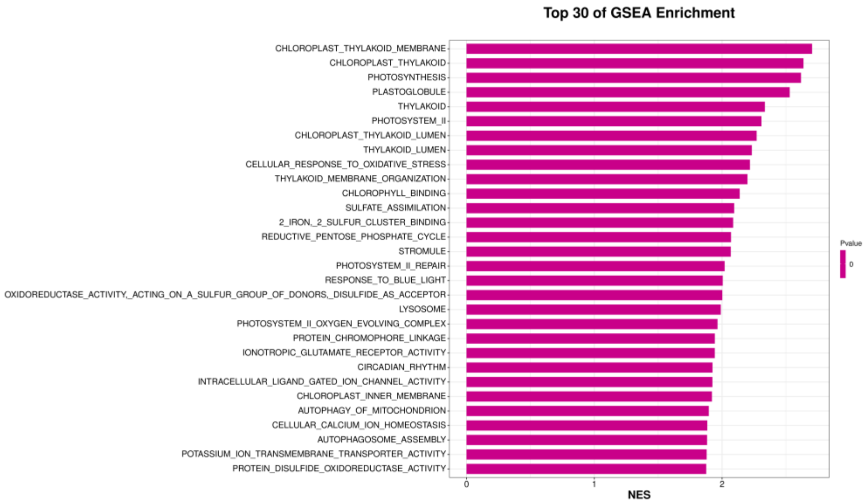

e.

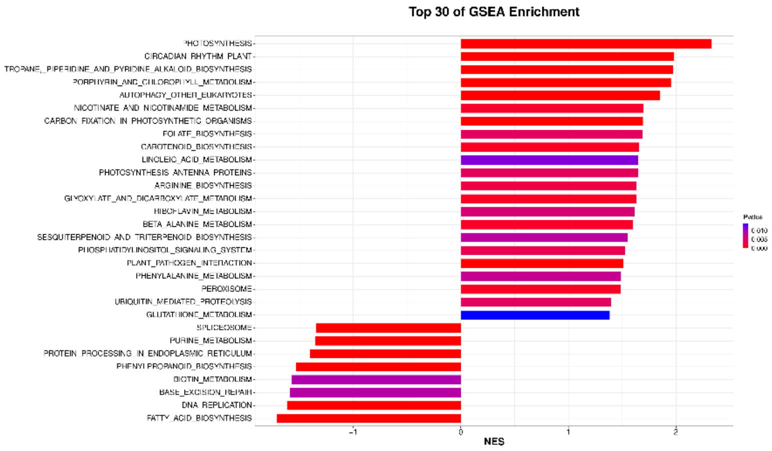

f.

Supplemental Figure S13. The volcano plot of gene expression pattern (a), the GO enrichment bar plot (b), the GO enrichment scatter plot (c), the KEGG enrichment scatter plot (d), the GO Gsea enrichment analysis (e) and the KEGG Gsea enrichment analysis (f) of DEGs between leaf tissue B and flower bud tissue E group.

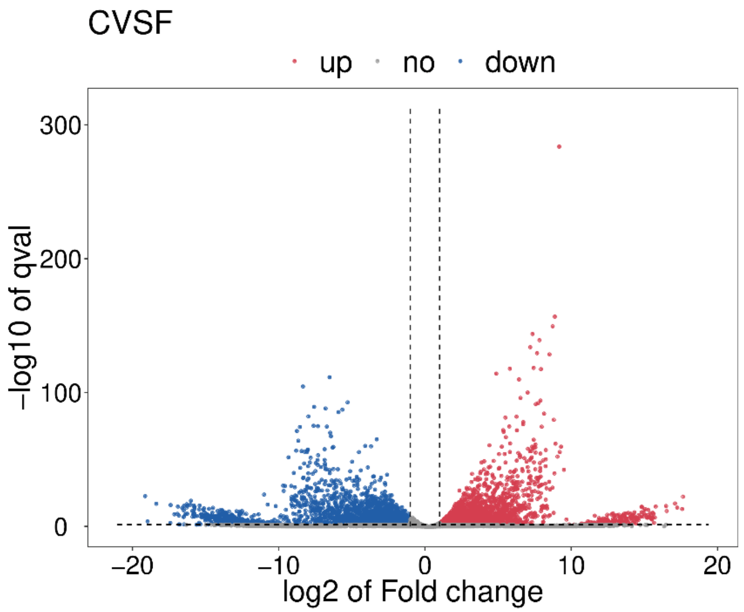

CVSF
up
no
down
300
200
100
0
−log10 of qval
−20
−10
0
10
20
log2 of Fold change

a.

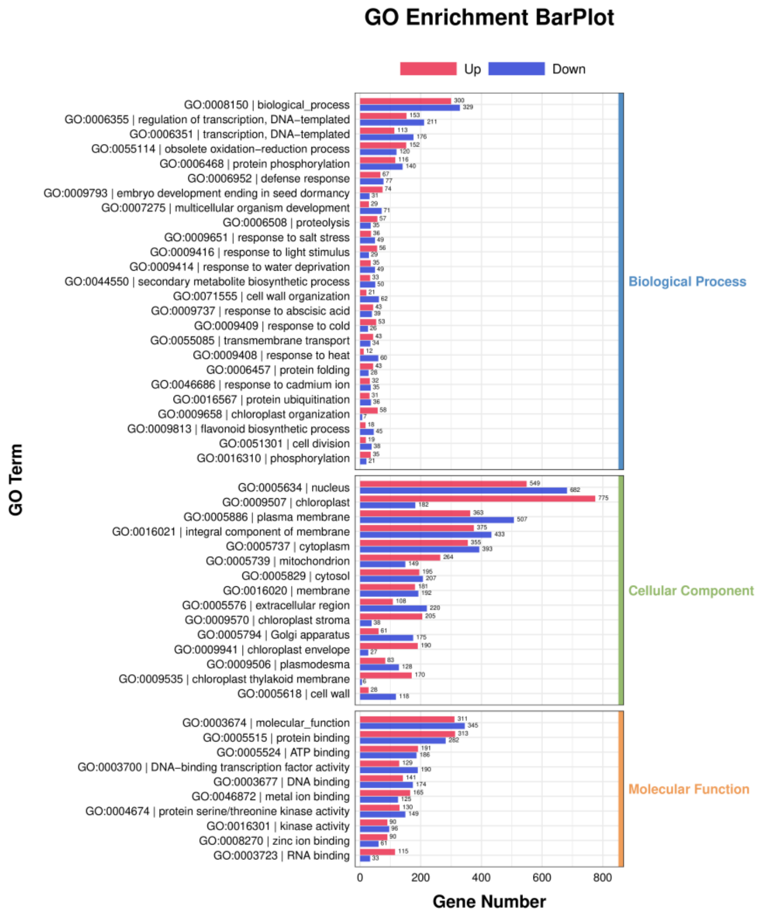

GO Enrichment BarPlot
Up
Down
GO Term
GO:0008150 | biological_process
GO:0006355 | regulation of transcription, DNA−templated
GO:0006351 | transcription, DNA−templated
GO:0055114 | obsolete oxidation−reduction process
GO:0006468 | protein phosphorylation
GO:0006952 | defense response
GO:0009793 | embryo development ending in seed dormancy
GO:0007275 | multicellular organism development
GO:0006508 | proteolysis
GO:0009651 | response to salt stress
GO:0009416 | response to light stimulus
GO:0009414 | response to water deprivation
GO:0044550 | secondary metabolite biosynthetic process
GO:0071555 | cell wall organization
GO:0009737 | response to abscisic acid
GO:0009409 | response to cold
GO:0055085 | transmembrane transport
GO:0009408 | response to heat
GO:0006457 | protein folding
GO:0046686 | response to cadmium ion
GO:0016567 | protein ubiquitination
GO:0009658 | chloroplast organization
GO:0009813 | flavonoid biosynthetic process
GO:0051301 | cell division
GO:0016310 | phosphorylation
Biological Process
GO:0005634 | nucleus
GO:0009507 | chloroplast
GO:0005886 | plasma membrane
GO:0016021 | integral component of membrane
GO:0005737 | cytoplasm
GO:0005739 | mitochondrion
GO:0005829 | cytosol
GO:0016020 | membrane
GO:0005576 | extracellular region
GO:0009570 | chloroplast stroma
GO:0005794 | Golgi apparatus
GO:0009941 | chloroplast envelope
GO:0009506 | plasmodesma
GO:0009535 | chloroplast thylakoid membrane
GO:0005618 | cell wall
Cellular Component
GO:0003674 | molecular_function
GO:0005515 | protein binding
GO:0005524 | ATP binding
GO:0003700 | DNA−binding transcription factor activity
GO:0003677 | DNA binding
GO:0046872 | metal ion binding
GO:0004674 | protein serine/threonine kinase activity
GO:0016301 | kinase activity
GO:0008270 | zinc ion binding
GO:0003723 | RNA binding
Molecular Function
0
200
400
600
800
Gene Number

b.

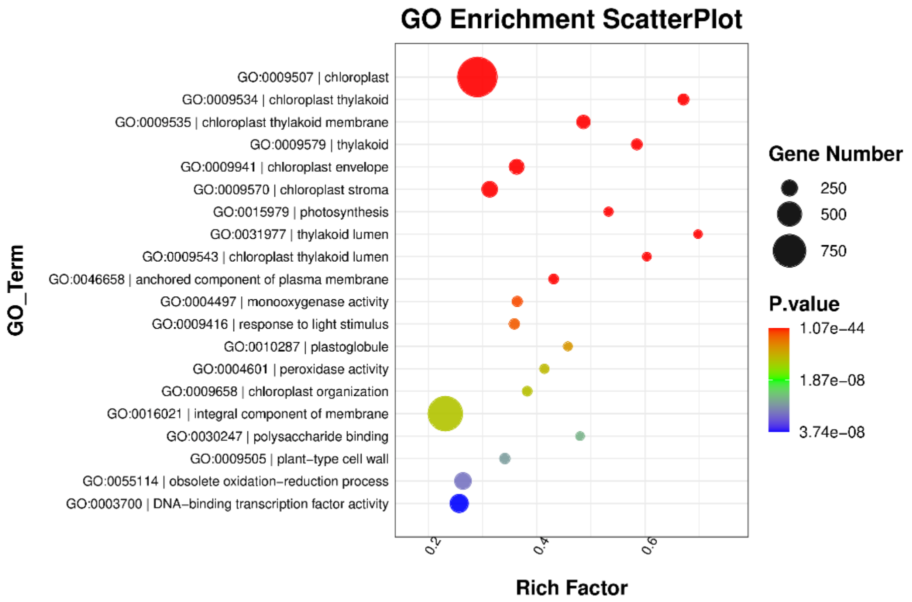
GO Enrichment ScatterPlot
GO:0009507 | chloroplast
GO:0009534 | chloroplast thylakoid
GO:0009535 | chloroplast thylakoid membrane
GO:0009579 | thylakoid
GO:0009941 | chloroplast envelope
GO:0009570 | chloroplast stroma
GO:0015979 | photosynthesis
GO:0031977 | thylakoid lumen
GO:0009543 | chloroplast thylakoid lumen
GO:0046658 | anchored component of plasma membrane
GO:0004497 | monooxygenase activity
GO:0009416 | response to light stimulus
GO:0010287 | plastoglobule
GO:0004601 | peroxidase activity
GO:0009658 | chloroplast organization
GO:0016021 | integral component of membrane
GO:0030247 | polysaccharide binding
GO:0009505 | plant-type cell wall
GO:0055114 | obsolete oxidation-reduction process
GO:0003700 | DNA-binding transcription factor activity
GO_Term
0.2
0.4
0.6
Rich Factor
Gene Number
250
500
750
P.value
1.07e−44
1.87e−08
3.74e−08

c.

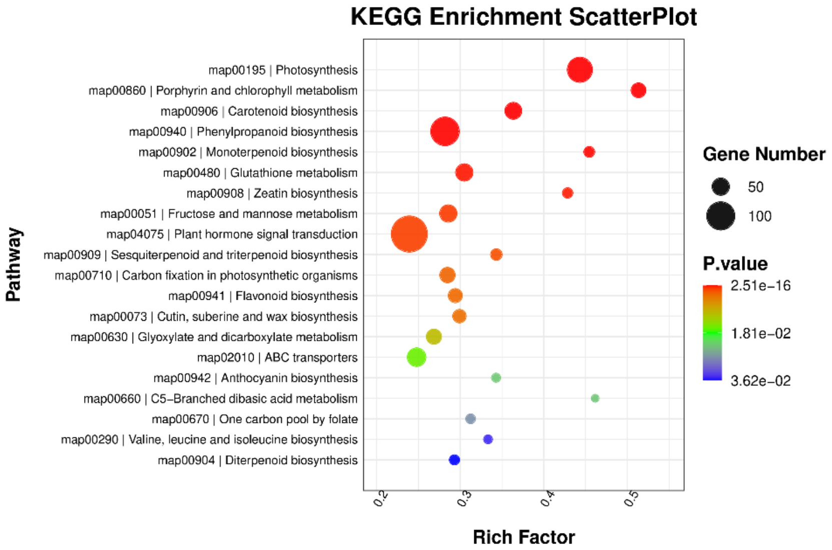
KEGG Enrichment ScatterPlot
map00195 | Photosynthesis
map00860 | Porphyrin and chlorophyll metabolism
map00906 | Carotenoid biosynthesis
map00940 | Phenylpropanoid biosynthesis
map00902 | Monoterpenoid biosynthesis
map00480 | Glutathione metabolism
map00908 | Zeatin biosynthesis
map00051 | Fructose and mannose metabolism
map04075 | Plant hormone signal transduction
map00909 | Sesquiterpenoid and triterpenoid biosynthesis
map00710 | Carbon fixation in photosynthetic organisms
map00941 | Flavonoid biosynthesis
map00073 | Cutin, suberine and wax biosynthesis
map00630 | Glyoxylate and dicarboxylate metabolism
map02010 | ABC transporters
map00942 | Anthocyanin biosynthesis
map00660 | C5−Branched dibasic acid metabolism
map00670 | One carbon pool by folate
map00290 | Valine, leucine and isoleucine biosynthesis
map00904 | Diterpenoid biosynthesis
Pathway
0.2
0.3
0.4
0.5
Rich Factor
Gene Number
50
100
P.value
2.51e−16
1.81e−02
3.62e−02

d.

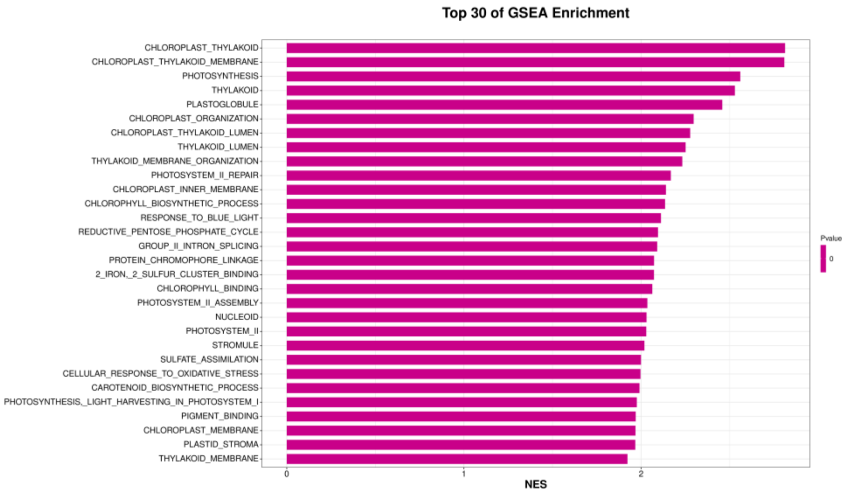

e.

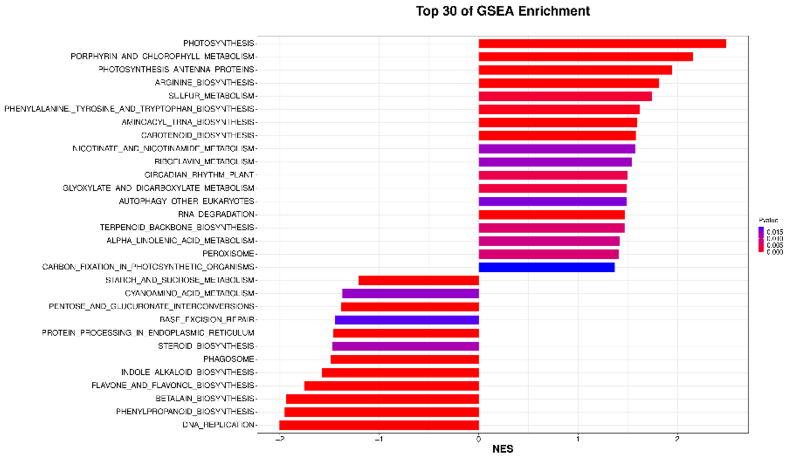

f.

Supplemental Figure S14. The volcano plot of gene expression pattern (a), the GO enrichment bar plot (b), the GO enrichment scatter plot (c), the KEGG enrichment scatter plot (d), the GO Gsea enrichment analysis (e) and the KEGG Gsea enrichment analysis (f) of DEGs between leaf tissue C and flower bud tissue F group.
